# Drinking water source and exposure to regulated water contaminants in the California Teachers Study cohort

**DOI:** 10.1038/s41370-024-00703-9

**Published:** 2024-07-13

**Authors:** Maya Spaur, Danielle N. Medgyesi, Komal Bangia, Jessica M. Madrigal, Lauren M. Hurwitz, Laura E. Beane Freeman, Jared A. Fisher, Emma S. Spielfogel, James V. Lacey, Tiffany Sanchez, Rena R. Jones, Mary H. Ward

**Affiliations:** 1 Occupational and Environmental Epidemiology Branch, Division of Cancer Epidemiology and Genetics, National Cancer Institute, Rockville, MD USA; 2 Department of Environmental Health Sciences, Mailman School of Public Health, Columbia University, New York, NY USA; 3 Community and Environmental Epidemiology Research Branch, Office of Environmental Health Hazard Assessment, Oakland, CA USA; 4 Division of Health Analytics, Department of Computational and Quantitative Medicine, Beckman Research Institute City of Hope, Duarte, CA USA

**Keywords:** Metals, Disinfection by-products, Volatile organic compounds, Exposure modeling, Environmental justice

## Abstract

**Background:**

Pollutants including metals/metalloids, nitrate, disinfection byproducts, and volatile organic compounds contaminate federally regulated community water systems (CWS) and unregulated domestic wells across the United States. Exposures and associated health effects, particularly at levels below regulatory limits, are understudied.

**Objective:**

We described drinking water sources and exposures for the California Teachers Study (CTS), a prospective cohort of female California teachers and administrators.

**Methods:**

Participants’ geocoded addresses at enrollment (1995–1996) were linked to CWS service area boundaries and monitoring data (*N* = 115,206, 92%); we computed average (1990–2015) concentrations of arsenic, uranium, nitrate, gross alpha (GA), five haloacetic acids (HAA5), total trihalomethanes (TTHM), trichloroethylene (TCE), and tetrachloroethylene (PCE). We used generalized linear regression to estimate geometric mean ratios of CWS exposures across demographic subgroups and neighborhood characteristics. Self-reported drinking water source and consumption at follow-up (2017–2019) were also described.

**Results:**

Medians (interquartile ranges) of average concentrations of all contaminants were below regulatory limits: arsenic: 1.03 (0.54,1.71) µg/L, uranium: 3.48 (1.01,6.18) µg/L, GA: 2.21 (1.32,3.67) pCi/L, nitrate: 0.54 (0.20,1.97) mg/L, HAA5: 8.67 (2.98,14.70) µg/L, and TTHM: 12.86 (4.58,21.95) µg/L. Among those who lived within a CWS boundary and self-reported drinking water information (2017–2019), approximately 74% self-reported their water source as municipal, 15% bottled, 2% private well, 4% other, and 5% did not know/missing. Spatially linked water source was largely consistent with self-reported source at follow-up (2017–2019). Relative to non-Hispanic white participants, average arsenic, uranium, GA, and nitrate concentrations were higher for Black, Hispanic and Native American participants. Relative to participants living in census block groups in the lowest socioeconomic status (SES) quartile, participants in higher SES quartiles had lower arsenic/uranium/GA/nitrate, and higher HAA5/TTHM. Non-metropolitan participants had higher arsenic/uranium/nitrate, and metropolitan participants had higher HAA5/TTHM.

**Impact:**

Though average water contaminant levels were mostly below regulatory limits in this large cohort of California women, we observed heterogeneity in exposures across sociodemographic subgroups and neighborhood characteristics. These data will be used to support future assessments of drinking water exposures and disease risk.

## Introduction

Drinking water represents an important source of exposure to inorganics (e.g., arsenic and nitrate), radionuclides (uranium, alpha particles), disinfection byproducts (DBPs), and volatile organic compounds (VOCs) for populations in the United States (U.S.) and worldwide [1]. Approximately 90% of the U.S. population is served by public water systems, and 10% by private wells [2]. In California, approximately 95% of the population is served by public water systems [3]. Public water systems include at least 15 service connections or serve at least 25 people; community water systems (CWS) are a type of public water system that serve the same population year-round [2]. Public water systems are regulated by the U.S. Environmental Protection Agency (EPA) under the Safe Drinking Water Act [4]. The contaminants we evaluated are regulated through federally enforceable maximum contaminant levels (MCLs), which were determined based on economic and technical feasibility, treatment technologies, cost-benefit analysis, and public health benefit for specific health endpoints [4]. States generally have primacy over enforcement of federal drinking water regulations. Notably, the MCL goal, a non-enforceable standard based solely on risk to health, is 0 µg/l for arsenic, uranium, alpha particles, trichloroethylene (TCE), tetrachloroethylene (PCE), bromodichloromethane, bromoform, and dichloroacetic acid, as there is no known safe level of exposure to these contaminants [4]. Private wells are not federally regulated or monitored.

Nitrate is a common contaminant of drinking water supplies in agricultural areas, due to use of nitrogen fertilizers and concentrated animal feeding operation waste [5, 6]. Atmospheric deposition, erosion of natural deposits, and septic tank or sewage leakage contribute to nitrate contamination in rural and urban areas [4]. Geogenic arsenic occurs in groundwater across the U.S., with regional differences due to climatic and geological factors; arid climates can cause evaporative concentration of arsenic in shallow groundwater supplies and lead to high levels, such as in the San Joaquin Valley of California [7–10]. Mining and historical arsenical pesticide use are anthropogenic sources of arsenic contamination in water supplies [8]. Uranium is present in different rock types and is leached from host mineral phases to surface and ground water supplies; uranium mining/milling and mobilization of uranium via nitrate fertilizer use are anthropogenic sources of uranium contamination [11–15]. Uranium and other radionuclides can decay and release alpha radiation, often quantified as total gross alpha for monitoring compliance purposes. DBPs are formed by the reaction of chlorine and bromine with natural organic compounds during the disinfection of water supplies to treat pathogens [16]. DBPs are commonly found in public water supplies across the U.S., with the highest concentrations observed in those reliant on surface water or shallow groundwater [16]. While over 700 DBPs have been identified, the most abundant classes are trihalomethanes (THMs, which include the chemicals chloroform, dibromochloromethane, bromodichloromethane, and bromoform, and regulated as the sum total, TTHM), and haloacetic acids (HAA5, regulated as the sum of dichloroacetic acid, trichloroacetic acid, monochloroacetic acid, bromoacetic acid, and dibromoacetic acid) [4, 17, 18]. The VOCs TCE and PCE are solvents used in dry cleaning, metal degreasing, textile, art, and industrial processes, and may be found in some consumer products [19]. Toxic waste disposal sites, sometimes recognized as Superfund sites under the Comprehensive Environmental Response, Compensation, and Liability Act (CERCLA), are anthropogenic sources of inorganic arsenic, uranium, TCE, and PCE in groundwater [12, 19–24].

Numerous studies implicate one or more of these drinking water contaminants in adverse health effects, including cancer, cardiovascular disease, reproductive and developmental toxicity, nephrotoxicity, and other adverse health conditions [1, 16, 20, 25–39]. Inorganic arsenic is classified by the International Agency for Research on Cancer (IARC) as a cause of cancers of the bladder, lung, and skin, and is associated with increased risk of cancers of the kidney, liver, and prostate [26]. Inorganic arsenic is also a potent toxicant associated with numerous adverse health outcomes, including cardiovascular disease, hypertension, and reproductive disorders [26, 31, 32]. Uranium exposure through drinking water is associated with renal damage and nephrotoxicity, and an increased risk of colorectal, breast, kidney, prostate, and total cancer [20, 27, 33]. Nitrate is classified by IARC as a probable human carcinogen when ingested under conditions that result in the endogenous formation of N-nitroso-compounds, most of which are animal carcinogens [28, 34]. Cancers of the stomach, colon, bladder, kidney, ovary, and thyroid, and thyroid disease are associated with elevated nitrate ingested from drinking water; however, the number of studies of most cancer sites is limited [29, 35]. Higher intake of DBPs through drinking water is associated with increased bladder cancer risk, and a limited number of studies suggest DBP exposures are potential risk factors for colon, rectum, and endometrial cancer [16, 36]. TCE is classified as carcinogenic to humans based on kidney cancer, and PCE (Group 2A) as probably carcinogenic to humans based on bladder cancer evidence [30]. Occupational studies also support adverse developmental, neurological, and hepatotoxic effects of TCE and PCE exposures [19]. Assessment of long-term drinking water contaminant exposures and associated health risks have traditionally been limited by the lack of water quality data that could be assigned to individuals in epidemiologic cohorts; understanding large-scale water quality data at the level of consumer intake is a critical research gap [40]. Additionally, there are relatively few cohort studies evaluating drinking water exposures at levels below the MCLs and World Health Organization guidelines that are commonly experienced by the general U.S. population [1]. Inequalities in CWS arsenic, uranium, and nitrate exposures by sociodemographic characteristics such as, race and ethnicity, income, education, region, and rurality/urbanicity have been documented [41, 42]. Few studies have evaluated sociodemographic inequalities in DBP and TCE/PCE exposures in the United States.

Our primary objective for this study was to describe exposure to regulated, frequently detected and measured contaminants in drinking water in the California Teachers Study (CTS), a large prospective cohort of women. We described the spatial linkage of participants’ residences to their drinking water source and corresponding estimates of contaminant concentrations. For a subset, we evaluated the agreement between address-assigned and self-reported drinking water source and described the daily intake of tap water and CWS contaminants. Additionally, we examined inequalities in CWS exposures across sociodemographic groups.

## Materials and methods

### Study population

We included participants from the CTS, a prospective cohort of women designed to investigate the etiology of breast and other cancers [43]. The CTS was originally designed to study higher rates of breast and other cancers observed in female teachers. Female public school teachers and administrators enrolled in the California State Teachers Retirement System were mailed a self-administered questionnaire, and 133,477 completed the questionnaire and joined the cohort in 1995–1996 (Supplementary Fig. S1) [43]. Of those, 124,685 had valid California addresses at the time of enrollment. Participants provided information on sociodemographic characteristics (race and ethnicity, age, education), anthropometrics (height and weight), smoking and alcohol consumption, and personal and family medical history. Participant race and ethnicity were categorized at enrollment as follows: non-Hispanic white (only white reported), Black (Black only or white and Black reported), Hispanic (Hispanic only or white and Hispanic reported), Native American (Native American only or white and Native American reported), Asian/Pacific Islander (Asian/Pacific Islander only [Chinese, Filipino, Hawaiian, Japanese, Vietnamese, or Korean] or white and Asian/Pacific Islander), or Other/multi-racial (Other reported or more than one of the aforementioned groups reported).

### Drinking water data and linkages

Our study used a geospatial dataset of statewide drinking water boundaries from the Water Boundary Tool (version updated in 2019), created by the Public Health Institute’s California Environmental Heath Tracking Program (CEHTP) [44, 45]. The California Office of Environmental Health Hazard Assessment (OEHHA) then cleaned and processed the geospatial layer by repairing geometry and reconciling overlaying boundaries. The OEHHA dataset includes CWS service areas collected previously by water system operators and local regulatory agencies; for areas outside of CWS boundaries, regions were partitioned into Public Land Survey Section (PLSS) sections, approximately 1 × 1 miles, and ambient groundwater contaminant concentrations were estimated (described below) [46]. For 124,665 eligible and consenting participants with a geocoded address at enrollment in California (Texas A&M, USC Geocoding Platform) [47], we linked their addresses to drinking water boundaries using QGIS [44]. Participants whose geocodes did not intersect a water boundary (*N* = 36) were manually assigned to the nearest water boundary. Most participants lived within a CWS service area (*N* = 115,206 (92%); CWS *N* = 1249) and were assumed to be using the corresponding CWS as their residential water source; CWS serving CTS participants were located across California (Fig. S2). The CWS for Los Angeles City was subdivided into five subsections based on their source of water and treatment plants. The remaining participants with addresses located outside of a CWS service area (*N* = 9459; 8%) were assumed to be served by domestic wells (systems that serve less than 5 service connections) or state small water systems (5–14 connections), which are not regulated or monitored by California.

CWS-level monitoring data (1990–2020) were obtained from and processed by OEHHA [44, 47]. Average annual concentrations of each contaminant were computed for each CWS as follows. We prioritized contaminants measured in samples of treated/delivered drinking water. When treated samples were not available for a contaminant, we averaged results from raw/untreated samples [44]. Only samples collected from CWS during active periods of use were included. Uranium concentrations were converted from pCi/l to µg/l using 1.49 as the conversion factor (pCi/l*1.49 = µg/l) [48]. CWS are required to report non-detections and concentrations based on the detection limits for the purposes of reporting (DLR) (Table S1), which is often higher than the laboratory limit of quantification. When samples had concentrations of zero, were reported as equal to, half of, or below the DLR, concentrations were imputed using Tobit regression based on the existing measurement data and assuming a log-normal distribution [49]. The upper bound for imputation was contaminant specific and was derived from the median of reported concentrations below the DLR (Table S1); the lower bound was zero. Among CWS matched to participants’ enrollment addresses, the population-weighted average percent of years (1990–2020) that CWS reported detectable levels of contaminants was 86% for gross alpha, 80% for uranium, 83% for nitrate-nitrogen (N) (hereafter referred to as nitrate), 88% for HAA5, 70% for TTHM, 64% for arsenic, 28% for TCE, and 35% for PCE (Table S2). Fewer CWS reported measurement data for uranium and HAA5 compared to the number of CWS that reported data for the other contaminants; years with missing measurement data were not included in the computation of average concentrations.

Domestic well estimates were computed by OEHHA using groundwater measurement data collected from 2011–2019 in the Groundwater Ambient Monitoring and Assessment Program, and were available at the Public Land Survey System level [44, 50]. Briefly, each section was assigned the average groundwater quality data from all wells within the section. Non-domestic wells were limited to those with well depths similar to domestic wells. Sections with no ambient groundwater quality data were assigned the average of all neighboring sections’ data. In cases where all neighboring sections had no data, the section was assigned the average ambient groundwater quality data from the township (6 × 6 miles) [51]. Domestic well data below the limits of detection were not imputed.

### Self-reported drinking water source and consumption at questionnaire 6

In the CTS questionnaire 6 (2017–2019, *N* = 39,031, 34% response rate), participants were asked their drinking water source at the current home (municipal water, private well, bottled water, other, don’t know), and whether the current home tap water was filtered/treated (which could include filtering pitchers such as Brita®, but did not include water softeners) [52]. Participants were also asked the number of glasses of water, cups of coffee, and cups of tea, usually consumed per day (made with household tap water) with responses of “Never”, “1 cup [or glass] a day”, “2 cups a day” up to “6+ cups a day”. The latter was coded as 6; responses of “Occasionally, i.e., not every day,” were coded as 0.5; responses of “Never” and “Skipped” were coded as zero. We treated responses of “Drank but don’t know how much” as missing (*N* = 491). We converted the number of cups per day to liters (l) per day assuming that a glass contained 12 ounces [53]. We linked the addresses of participants who completed questionnaire 6 to a CWS service boundary or PLSS section. Among participants that were linked to a CWS and self-reported using municipal water, we calculated the daily contaminant intake from tap water using the following equation: (Contaminant concentration*Intake Rate [l/day]). We calculated the intake rate for tap water only (*N* = 22,067 with self-reported tap water consumption) and total water including coffee and tea (*N* = 20,657 with self-reported tap water consumption of water, coffee, and tea). We calculated the contaminant concentration using monitoring data from 2016–2020, to align with the time frame of questionnaire 6.

A subset of participants with a self-reported municipal water source were able to provide the name of the water company at their current home (*N* = 9834) [54]. We compared the agreement of the self-reported and the assigned CWS name, to evaluate the accuracy of our linkage-based exposure assessment. First, we converted the water system names to lowercase and removed blank spaces. We then calculated the Levenshtein distance between the CWS names to assess similarity between the strings (we defined a match as strings with Levenshtein distance ≤5 based on visual assessment of different distances), matched by the first letter and partial common components of the strings, and performed a manual check to correct matches and non-matches that were inaccurately classified using the automated processes.

### Census tract covariates

A neighborhood socioeconomic status (SES) metric for the enrollment address was previously created for CTS participants based on the enrollment address and incorporating three 1990 census block group variables [55]. Briefly, quartile values were computed based on the statewide estimates of educational attainment (percentage of adults over age 25 completing a college degree or higher), income (median family income), and occupation (half of adults employed in managerial/professional occupations). Census block groups were assigned a score of 1 (low) through 4 (high) for each of the SES attributes. Scores were summed across the attributes and census block groups were categorized into four groups based on quartiles of this overall score [55].

Urbanicity of the enrollment address was also previously characterized based on information from the 1990 U.S. Census [55]. Metropolitan urban area was defined as the highest quartile of population density within an Urbanized Area (population ≥ 1,000,000) and Metropolitan suburban area was defined as the rest of the population (lower three quartiles) within an Urbanized Area (population ≥ 1,000,000). City was defined as Census Places outside of an Urbanized Area with a population ≥50,000 people, and Town was defined as Census Places outside of an Urbanized Area with a population <50,000 people and in the upper three quartiles of population density. Rural was defined as Census Places outside of an Urbanized Area with <50,000 people and in the lowest quartile of population density, and unpopulated areas (Table S3) [55]. We dichotomized urbanicity into metropolitan areas (metropolitan urban and metropolitan suburban) and non-metropolitan areas (city, town, rural).

### Statistical analyses

All statistical analyses were conducted in R version 4.0.2 within the CTS Researcher Platform [56]. We described participant characteristics by water source at enrollment. Because most participants were served by CWS, we did not conduct further analysis of the domestic well exposures. We estimated long-term averages of CWS contaminants at the enrollment address (1990–2015), and the percent of years that annual average concentrations were ≥half the MCL and ≥MCL, out of the total number of years of measurement data reported per CWS in the 1990–2015 period. To align with the timing of self-reported information on residential drinking water source at questionnaire 6 (2017–2019), we described 2016–2020 average CWS concentrations at the questionnaire 6 address, among participants who self-reported drinking municipal water.

Residential duration at the enrollment address was previously estimated for CTS participants [57]. Briefly, addresses from enrollment (1995–1996) through 2019 were obtained from participants who completed follow-up questionnaires, as well as from the U.S. Postal Service, LexisNexis, Experian, and the California Cancer Registry databases [55]. The mean (median) total residential duration at the enrollment address was 22.2 (22.7) years. As a sensitivity analysis, we computed long-term average exposures restricted to participants residing at the enrollment address for at least 20 years before and/or after enrollment (*N* = 60,972).

We performed Spearman correlation analyses to describe correlations between contaminants. We compared CWS exposures by sociodemographic subgroups: participant race and ethnicity, and census block group-level SES quartile and urbanicity. We used generalized linear regression to estimate unadjusted and adjusted geometric mean ratios (GMRs) of CWS exposures by race and ethnicity (non-Hispanic white as reference), census block group SES quartile (quartile 1 as reference), and urbanicity (non-metropolitan areas as reference); adjusted analyses were co-adjusted for the other two sociodemographic variables. Finally, we compared self-reported drinking water source to the spatially linked drinking water source (CWS or domestic well) at questionnaire 6. We evaluated the percent agreement between participants’ self-reported water company name and the spatially linked, standardized CWS name, as described above. Among participants self-reporting municipal tap water as their drinking water source, we computed daily tap water and CWS contaminant intake.

In supplemental analyses, we described temporal trends in CWS-level contaminant concentrations, among the 1249 CWS linked to CTS participants at enrollment. We used generalized linear regression models to estimate GMRs of 10-year average CWS concentrations (1990–1999 average concentration as reference), adjusting for water source type (groundwater, groundwater under the influence of surface water, surface water) and population served (very small (≤500 people), small (>500–3300 people), medium (>3300–10,000 people), large (>10,000–<1,000,000 people), and very large (≥1,000,000 people)). Due to limited HAA5 data prior to 2000, we compared average HAA5 concentrations in 2000–2009 (reference) to the average concentration in 2010–2020.

## Results

### Descriptive characteristics by enrollment drinking water source

We included 125,665 CTS participants with a valid California residential address at the time of enrollment and who had consented to non-breast cancer research. Median age at enrollment (1995–1996) was 52 years old with almost all (99%) participants having attained a bachelor’s degree or higher degree (Table 1). A majority of all participants were non-Hispanic white (87%), followed by Hispanic (4%), Asian (4%), Black (3%), other/multi-racial (1%), and Native American (1%). By water source, 80% of CWS users and 50% of domestic well users lived in census block groups in the upper two quartiles of SES. While 86% of domestic well users lived in non-metropolitan areas, 32% of CWS users lived in non-metropolitan areas, and 68% lived in metropolitan areas (Table 1). Comparing CWS and domestic well exposures, average nitrate concentrations were more than twice as high among domestic well users (median = 1.25 mg/l) compared to CWS users (median = 0.54 mg/l); median arsenic and gross alpha concentrations were higher among CWS users (1.03 µg/l and 2.21 pCi/l, respectively) compared to domestic well users (0.62 µg/l and 0.38 pCi/l, respectively), though the 75th percentile of arsenic concentrations were higher among domestic well users. Uranium concentration estimates were not available for domestic well users. TTHM, HAA5, TCE, and PCE exposures occurred mostly among those using CWS (median (µg/l) of HAA5 = 8.67, TTHM = 12.86, TCE = 0.01, PCE = 0.02), compared to domestic well users (median = 0 µg/l).

**Table 1:** Characteristics of California Teachers Study (CTS) participants, by drinking water source (community water system, CWS, and domestic well) assigned by enrollment address.

	CWS, *N* = 115,206	Domestic well, *N* = 9459
Age, years, Median (IQR)	52 (43,64)	52 (44,64)
BMI, kg/m^2^, Median (IQR)	23.6 (21.3,27.2)	23.8 (21.5,27.4)
Race and ethnicity, *N* (%)		
Non-Hispanic white	99,223 (86)	8659 (92)
Black	3277 (3)	55 (1)
Hispanic	5024 (4)	244 (3)
Native American	1035 (1)	115 (1)
Asian	4180 (4)	184 (2)
Other/multi-racial	1423 (1)	120 (1)
None reported	1044 (1)	82 (1)
Smoking status, *N* (%)		
Never smoker	75,633 (66)	6356 (68)
Former smoker	32,953 (29)	2587 (28)
Current smoker	5846 (5)	447 (5)
Alcohol consumption, *N* (%)		
0 g/day alcohol	36,957 (34)	3021 (34)
<20 g/day alcohol	62,856 (58)	5135 (57)
≥20 g/day alcohol	8946 (8)	849 (9)
Education, *N* (%)		
<High School	1 (0)	0 (0)
High School, Technical or Associate’s Degree	499 (1)	59 (1)
Bachelor’s	24,259 (39)	2750 (52)
Master’s	33,064 (53)	2207 (41)
Doctoral^a^	4343 (7)	299 (6)
SES quartile^b^, *N* (%)		
Q1 (Low SES block group)	4392 (4)	1111 (13)
Q2	18,277 (16)	3257 (37)
Q3	37,486 (33)	2821 (32)
Q4 (High SES block group)	54,028 (47)	1618 (18)
Urbanicity^c^, *N* (%)		
Not metropolitan	36,432 (32)	7563 (86)
Metropolitan	77,797 (68)	1250 (14)
Average water concentration, Median (IQR)^d^		
Arsenic (µg/l)	1.03 (0.54, 1.71)	0.62 (0, 3.24)
Uranium (µg/l)	3.48 (1.01, 6.18)	–^e^
Gross alpha (pCi/l)	2.21 (1.32, 3.67)	0.38 (0, 3.19)
Nitrate-nitrogen (mg/l)	0.54 (0.20, 1.97)	1.25 (0.09, 3.38)
HAA5 (µg/l)	8.67 (2.98, 14.70)	0 (0, 1.23)
TTHM (µg/l)	12.86 (4.58, 21.95)	0 (0, 0.04)
TCE (µg/l)	0.01 (0.01, 0.16)	0 (0, 0)
PCE (µg/l)	0.02 (0.01, 0.10)	0 (0, 0)

^a^Doctoral degree includes PhD, EdD, MD, DDS, DVD, LLB, and JD.

^b^A summary SES metric was created incorporating three 1990 census block group variables (occupation, education, and income) (Hurley et al. [55]).

^c^Urbanization categories were created using 1990 census block groups (Hurley et al. [55]) and dichotomized as non-metropolitan (rural, town, city), and metropolitan (metropolitan suburban and metropolitan urban).

^d^CWS analyte concentrations represent averages of annual average concentrations from 1990–2015. CWS measurement data below the detection limit for the purposes of reporting (DLR) were imputed using Tobit regression. Domestic well concentrations were estimated from data collected from 2011–2019 (Groundwater Ambient Monitoring & Assessment, 2023) and were not imputed; zeros represent values below the detection limit. Average CWS estimates were available for the following *N* of participants: arsenic (114,794), uranium (111,174), gross alpha (114,709), nitrate (114,810), HAA5 (99,461), TTHM (113,480), TCE (114,789), and PCE (114,789). Average domestic well estimates were available for the following *N* of participants: arsenic (7930), gross alpha (7412), nitrate (8382), HAA5 (1328), TTHM (7067), TCE and PCE (7691).

^e^Uranium concentrations were not available for domestic wells.

In Spearman correlation analyses of CWS exposures, the participants’ long-term (1990–2015) average concentrations of uranium and gross alpha were highly correlated (rho = 0.85) (Fig. S3). More moderate (0.08–0.67) positive pairwise correlations were observed between nitrate, arsenic, uranium, gross alpha, TCE, and PCE. The summed classes of disinfection byproducts HAA5 and TTHM were positively correlated with each other (rho = 0.52), and negatively or negligibly correlated with all other contaminants.

CWS contaminant exposures are summarized as average concentrations from 1990–2015, and as the percent of years that annual average concentrations were ≥1/2 MCL and ≥MCL in that period (Table 2). Overall, the means and medians of the average concentrations were below regulatory limits for all contaminants, though there was considerable variability in ranges. Of note, fewer CWS reported measurement data in this period for uranium (CWS *N* = 860) and HAA5 (CWS *N* = 882) than the other contaminants [44]. Almost double the number of CWS reported HAA5 data in 2010–2020 (*N* = 957) compared to before 2010 (*N* = 480) (Fig. S4). Median and mean average concentrations and percent of years of data ≥1/2 MCL and ≥MCL were similar when we restricted analyses to participants that resided at their enrollment address for at least 20 years (pre and/or post enrollment) (Table S4).

**Table 2:** Distributions of long-term (1990–2015) average concentrations of community water system (CWS) exposures^a^, and percent of years the annual average concentration was at or above the maximum contaminant level (MCL) and half of the MCL^b^.

	**Average concentration**					
	***N*** **Participants**	***N*** **CWS**	**Mean (SD)**	**Median (25th, 75th)**	**95th %**	**Range**^**c**^
Arsenic (µg/l)	114,794	1217	1.44 (1.82)	1.03 (0.54,1.71)	3.97	0.01, 75.52
Uranium (µg/l)	111,174	860	4.49 (4.13)	3.48 (1.01,6.18)	12.63	0.03, 96.74
Gross alpha (pCi/l)	114,709	1207	2.76 (1.96)	2.21 (1.32,3.67)	6.36	0.18, 55.28
Nitrate-N (mg/l)	114,810	1225	1.31 (1.54)	0.54 (0.20,1.97)	5.08	0.02, 59.57
HAA5 (µg/l)	99,461	882	10.30 (8.61)	8.67 (2.98,14.70)	29.00	0.13, 115.18
TTHM (µg/l)	113,480	1220	14.63 (11.98)	12.86 (4.58,21.95)	36.51	0, 105.45
TCE (µg/l)	114,789	1216	0.27 (1.31)	0.01 (0.01,0.16)	0.74	0, 32.89
PCE (µg/l)	114,789	1216	0.24 (1.46)	0.02 (0.01,0.10)	0.66	0, 25.03
	**% of years** ≥ **1/2 MCL**			**% of years** ≥ **MCL**		
	***N*** **Participants**	***N*** **CWS**	**Mean (SD)**	***N*** **Participants**	***N*** **CWS**	**Mean (SD)**
Arsenic	37,236	384	5 (13)	12,557	177	1 (6)
Uranium	30,451	172	5 (13)	5475	55	1 (3)
Gross alpha	38,772	299	5 (13)	13,227	97	1 (2)
Nitrate-nitrogen	24,699	275	6 (16)	2769	44	0 (1)
HAA5	14,442	142	5 (16)	687	29	0 (1)
TTHM	69,979	377	13 (17)	4728	66	0 (2)
TCE	14,996	48	2 (8)	4853	20	1 (6)
PCE	15,126	66	2 (8)	7214	36	1 (4)

CWSs were assigned to CTS participants by enrollment address.

^a^Community water system arsenic, uranium, gross alpha, nitrate-nitrogen (N), five haloacetic acids (HAA5), total trihalomethanes (TTHM), trichloroethylene (TCE) and tetrachloroethylene (PCE) concentrations were assigned to California Teachers Study (CTS) participants by enrollment address.

^b^The MCLs are as follows: arsenic (10 µg/l), uranium (30 μg/l), gross alpha (15 pCi/l, not including radon and uranium), nitrate-N (10 mg/l), TTHM (80 μg/l), HAA5 (60 μg/l), TCE (5 μg/l), and PCE (5 μg/l). Measurement data below the detection limit for the purposes of reporting (DLR) were imputed using Tobit regression. % of years ≥½ MCL = total number of years that the annual average concentration was ≥½ MCL/total number of years of measurement data per CWS. % of years ≥MCL = total number of years that the annual average concentration was ≥MCL/total number of years of measurement data per CWS. *N* participants and *N* CWS indicate the *N* of participants and *N* CWS that had at least one annual average concentration ≥½ MCL or ≥MCL.

^c^Range = minimum, maximum.

### CWS exposures by sociodemographic factors

**Fig. 1:**
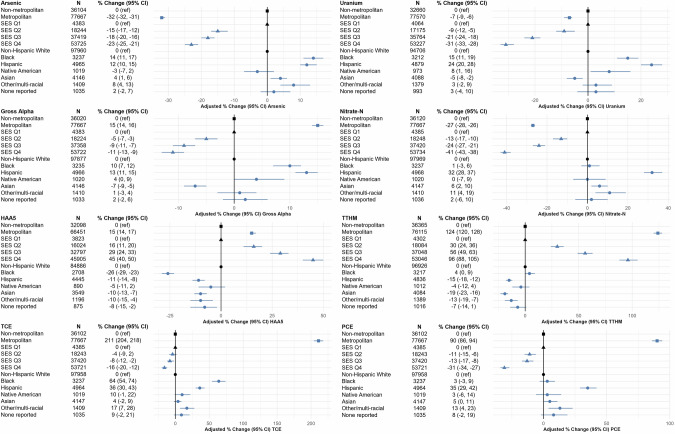
Adjusted percent (%) change (95% CI)^1^ of long-term (1990–2015) average concentrations of community water system (CWS) exposures^2^ by census block group-level socioeconomic status quartile (SES)^3^ and urbanicity^4^, and participant race and ethnicity (*N* = 114,183 participants with race/ethnicity, SES, and urbanicity information). Reference groups are in black, non-reference groups are in blue. Shapes represent each sociodemographic group category (square = urbanicity, triangle = SES, circle = race/ethnicity). ^1^Generalized linear regression was used to compute geometric mean ratios (GMRs) (95% confidence intervals, CI) of natural-log transformed exposure estimates by SES quartile (reference = SES quartile 1), urbanicity (reference = non-metropolitan areas), and participant race/ethnicity (reference = non-Hispanic white participants). Model was co-adjusted for SES, urbanicity, participant race/ethnicity. We calculated percent changes as: (GMR − 1) × 100%. ^2^Contaminants are as follows: arsenic (µg/l), uranium (μg/l), gross alpha (pCi/l), nitrate-nitrogen (Nitrate-N, mg/l), total trihalomethanes (TTHM, μg/l), five haloacetic acids (HAA5, μg/l), trichloroethylene (TCE, μg/l), and tetrachloroethylene (PCE, μg/l). Measurement data below the detection limit for the purposes of reporting (DLR) were imputed using Tobit regression. ^3^SES metric was created incorporating three 1990 census block group variables (occupation, education, and income) (Hurley et al. [55]). ^4^Urbanization categories were created using 1990 census block groups (Hurley et al. [55]) and dichotomized as non-metropolitan (rural, town, city), and metropolitan (metropolitan suburban and metropolitan urban).

We described average CWS exposures stratified by census block-group level SES quartile and urbanicity, and participant race and ethnicity (Table 3 and Figs. S5 and S6). In adjusted analyses (model 2), relative to non-Hispanic white participants, arsenic concentrations were 14% higher (95% CI 11, 17%), 12% higher (95% CI 10, 15%), and 8% higher (95% CI 4, 13%) for Black, Hispanic, and other/multi-racial participants, respectively (Fig. 1). Relative to non-Hispanic white participants, uranium was 15% higher (95% CI 11, 19%), 24% higher (95% CI 20, 28), and 8% higher (95% CI 1, 16%) for Black, Hispanic, and Native American participants, respectively, with similar patterns observed for gross alpha. Nitrate levels were 32% (95% CI 28, 37%) higher for Hispanic participants, 6% (95% CI 2, 10%) higher for Asian participants, and 11% (95% CI 4, 19%) higher for other/multi-racial participants. Relative to non-Hispanic white participants, TTHM levels were 4% (95% CI 0, 9%) higher for Black participants, but lower for all other racial/ethnic groups. Similarly, HAA5 levels were lower for all other racial/ethnic groups compared to non-Hispanic white participants. Conversely, TCE and PCE levels were elevated for all racial/ethnic groups compared to non-Hispanic white participants. Results were similar in univariate analyses (model 1, Fig. S7).

**Table 3:** Distributions^a^ of long-term (1990–2015) average concentrations of community water system (CWS) exposures by census block group-level socioeconomic status quartile (SES)^b^ and urbanicity^c^, and participant race and ethnicity.

	**Median (IQR), 95th percentile, by census block group-level SES quartile, urbanicity**						
**Concentration**	**SES Q1**	**SES Q2**	**SES Q3**	**SES Q4**	**Not metropolitan**	**Metropolitan**	
	***N*** = 4392	***N*** = 18,277	***N*** = 37,486	***N*** = 54,028	***N*** = 36,432	***N*** = 77,797	
Arsenic (µg/l)	1.43 (0.79,2.18), 6.59	1.13 (0.67,1.84), 5.02	1.07 (0.56,1.72), 4.54	1.00 (0.47,1.55), 3.11	1.49 (0.63,2.32), 6.59	0.99 (0.48,1.34), 2.77	
Uranium (µg/l)	4.43 (2.82,7.56), 14.12	4.02 (1.86,7.28), 14.12	3.65 (1.13,6.38), 12.63	3.21 (0.87,5.39), 12.35	3.62 (1.30,7.95), 14.12	3.43 (1.01,5.44), 10.69	
Gross alpha (pCi/l)	2.31 (1.50,3.97), 5.83	2.21 (1.27,3.70), 6.49	2.21 (1.24,3.67), 6.38	2.21 (1.42,3.67), 6.14	2.05 (1.18,3.59), 6.11	2.45 (1.50,3.77), 6.49	
Nitrate-N (mg/l)	1.43 (0.34,2.73), 5.39	1.02 (0.27,2.56), 5.39	0.74 (0.25,2.10), 4.88	0.37 (0.17,1.58), 4.64	1.03 (0.27,2.44), 5.51	0.40 (0.17,1.84), 4.36	
HAA5 (µg/l)	5.50 (1.33,12.39), 28.41	6.58 (1.85,13.53), 28.41	8.17 (2.79,14.04), 28.41	10.40 (4.17,15.23), 29.79	6.06 (1.80,14.07), 34.88	10.12 (4.17,14.86), 21.67	
TTHM (µg/l)	6.06 (0.91,17.29), 31.50	8.18 (1.88,19.46), 32.99	11.20 (4.28,20.70), 36.51	13.16 (6.84,23.71), 36.51	7.80 (0.86,19.16), 32.87	12.86 (6.09,22.58), 36.51	
TCE (µg/l)	0.01 (0.01,0.12), 0.74	0.01 (0.01,0.10), 0.74	0.01 (0.01,0.15), 0.74	0.01 (0.01,0.18), 0.74	0.01 (0.01,0.02), 0.43	0.02 (0.01,0.30), 0.86	
PCE (µg/l)	0.02 (0.01,0.16), 0.86	0.02 (0.01,0.14), 0.67	0.02 (0.01,0.13), 0.80	0.01 (0.01,0.10), 0.62	0.01 (0.01,0.07), 0.34	0.02 (0.01,0.16), 0.80	
	**Median (IQR), 95th percentile, by participant race and ethnicity**						
	**Non-Hispanic White**	**Black**	**Hispanic**	**Native American**	**Asian**	**Other/multi-racial**	**Not reported**
**Concentration**	***N*** = 99,223	***N*** = 3277	***N*** = 5024	***N*** = 1035	***N*** = 4180	***N*** = 1423	***N*** = 1044
Arsenic (µg/l)	1.03 (0.54,1.67), 3.82	1.00 (0.70,2.16), 3.11	1.14 (0.75,1.72), 4.17	1.10 (0.55,1.72), 3.65	1.00 (0.49,1.71), 4.17	1.07 (0.56,1.72), 4.01	1.03 (0.54,1.72), 4.17
Uranium (µg/l)	3.44 (1.01,6.18), 12.63	3.68 (2.29,7.28), 12.52	4.11 (2.52,6.47), 13.70	3.93 (1.43,7.08), 14.12	3.21 (0.80,6.17), 12.11	3.68 (1.01,6.38), 13.36	3.68 (1.01,6.47), 14.12
Gross alpha (pCi/l)	2.21 (1.26,3.67), 6.38	2.27 (1.55,4.28), 5.23	2.70 (1.73,3.77), 6.38	2.38 (1.42,3.77), 6.37	2.12 (1.23,3.67), 5.90	2.25 (1.50,3.67), 5.91	2.25 (1.40,3.70), 6.49
Nitrate-N (mg/l)	0.54 (0.20,1.96), 5.08	0.50 (0.23,2.10), 4.36	0.94 (0.29,2.69), 5.39	0.73 (0.20,2.04), 5.38	0.49 (0.22,2.10), 4.57	0.69 (0.22,2.25), 5.31	0.62 (0.20,2.10), 5.08
HAA5 (µg/l)	9.20 (3.02,14.86), 29.71	5.51 (1.18,14.04), 21.67	7.60 (2.01,13.46), 21.67	8.14 (2.53,13.59), 28.41	9.40 (1.67,15.37), 24.59	8.09 (1.84,14.04), 28.41	8.17 (2.04,14.04), 25.85
TTHM (µg/l)	12.86 (4.71,22.42), 36.51	12.86 (5.67,19.89), 31.50	10.10 (3.49,19.89), 36.51	12.30 (3.78,21.94), 36.51	12.45 (4.40,19.89), 33.26	11.88 (4.40,19.89), 36.51	12.86 (4.40,21.31), 36.51
TCE (µg/l)	0.01 (0.01,0.12), 0.73	0.03 (0.01,0.42), 0.74	0.02 (0.01,0.36), 0.99	0.01 (0.01,0.17), 0.73	0.01 (0.01,0.22), 0.92	0.02 (0.01,0.20), 0.75	0.01 (0.01,0.18), 0.74
PCE (µg/l)	0.01 (0.01,0.10), 0.62	0.05 (0.01,0.16), 0.52	0.03 (0.01,0.17), 1.30	0.02 (0.01,0.10), 0.66	0.02 (0.01,0.13), 1.69	0.02 (0.01,0.15), 0.95	0.02 (0.01,0.14), 0.62

^a^Distributions are presented as median (interquartile range [IQR: 25th percentile, 75th percentile]), 95th percentile. CWS exposures were assigned by enrollment address. Measurement data below the detection limit for the purposes of reporting (DLR) were imputed using Tobit regression.

^b^SES metric was created incorporating three 1990 census block group variables (occupation, education, and income) (Hurley et al. [55]).

^c^Urbanization categories were created using 1990 census block groups (Hurley et al. [55]) and dichotomized as non-metropolitan (rural, town, city), and metropolitan (metropolitan suburban and metropolitan urban).

### Self-reported drinking water source and characteristics

Among 38,865 participants who participated in questionnaire 6 (2017–2019) and completed the drinking water section, 33,276 participants (86%) resided in California and could be assigned to a water source (CWS or PLSS domestic well) based on their Q6 address. Seventy percent responded that their tap water source was municipal water, 6% reported private well water, 15% reported bottled water, 4% reported other, and 5% reported don’t know or had a missing response (Table 4). Based on the Q6 address, 30,737 participants (92%) were assigned as CWS users, while 2539 (8%) were assigned as domestic well users. Of participants spatially linked as CWS users, 74% responded that their tap water source was municipal water, 2% reported private well water, 15% reported bottled water, 4% reported other, and 5% reported don’t know or had a missing response. Among those assigned as domestic well users, 53% said they used a private well, 26% reported municipal water, 11% reported bottled water, 4% reported other, and 5% don’t know/missing.

**Table 4:** Comparisons of drinking water sources at the 6th survey (2017–2019): self-reported drinking water source vs. assigned water source^a^ (*N* = 33,276).

Self-reported water source, *N* (%)	Assigned water source, *N* (%)	
	CWS 30,737 (92)	Domestic well 2539 (8)
Municipal water		
23,356 (70)	22,687 (74)	669 (26)
Private well		
1927 (6)	582 (2)	1345 (53)
Bottled water		
5028 (15)	4744 (15)	284 (11)
Other		
1253 (4)	1141 (4)	112 (4)
Don’t know/missing		
1712 (5)	1583 (5)	129 (5)

Percentages represent column totals.

^a^Community water system (CWS) exposures were assigned to participants living within a CWS distribution boundary based on the address at questionnaire 6. Participants living outside of a distribution boundary were assigned as domestic well users.

The daily intakes of contaminants in tap water, based on the CWS 2016–2020 average concentrations, are described in Table 5. Daily intakes of each contaminant were calculated for participants who were assigned as CWS users and self-reported municipal tap water and intake of water, coffee, and tea. Median tap water intake including water, coffee, and tea was 2.01 l/day compared to 1.42 l/day for tap water alone. Mean daily intake from water, coffee, and tea was as follows: 1.97 µg arsenic/day, 6.85 µg uranium/day, 5.03 pCi/l gross alpha/day, 2.27 mg/l nitrate/day, 46.83 µg TTHM/day, 23.32 µg HAA5/day, 0.19 µg TCE/day, and 0.18 µg PCE/day (Table 5).

**Table 5:** Daily intake of community water system (CWS, 2016–2020) arsenic, uranium, gross alpha, nitrate-nitrogen (N), five haloacetic acids (HAA5), total trihalomethanes (TTHM), trichloroethylene (TCE) and tetrachloroethylene (PCE) per L of home tap water consumed per day.

	*N* Participants	Mean (SD)	Median (25th, 75th)	95th %	Range
Water^a^					
Total daily L water	22,067	1.31 (0.61)	1.42 (0.71,1.77)	2.13	0, 2.13
Arsenic (µg/day)	21,784	1.28 (1.84)	0.56 (0.29,1.49)	4.70	0, 21.36
Uranium (µg/day)	18,783	4.50 (5.70)	2.52 (0.92,5.71)	16.74	0, 151.69
Gross alpha (pCi/day)	20,532	3.31 (3.89)	2.16 (0.97,4.20)	10.29	0, 43.58
Nitrate-N (mg/day)	21,948	1.49 (2.26)	0.39 (0.12,2.06)	6.28	0, 16.08
TTHM (µg/day)	20,633	30.76 (25.03)	26.55 (9.50,45.96)	76.39	0, 288.56
HAA5 (µg/day)	18,378	15.29 (15.30)	10.65 (4.20,20.52)	47.38	0, 155.44
TCE (µg/day)	21,820	0.12 (0.90)	0.01 (0.01,0.03)	0.22	0, 19.32
PCE (µg/day)	21,820	0.12 (0.61)	0.01 (0.01,0.02)	0.45	0, 11.31
Water, coffee, tea^b^					
Total daily L water, coffee, tea	20,657	2.00 (0.75)	2.01 (1.54,2.48)	3.13	0, 7.10
Arsenic (µg/day)	20,399	1.97 (2.73)	0.90 (0.45,2.40)	6.87	0, 51.70
Uranium (µg/day)	17,579	6.85 (8.45)	3.99 (1.57,8.67)	23.94	0, 311.82
Gross alpha (pCi/day)	19,214	5.03 (5.57)	3.41 (1.65,6.24)	15.16	0, 94.42
Nitrate-N (mg/day)	20,549	2.27 (3.29)	0.63 (0.18,3.23)	9.21	0, 28.09
TTHM (µg/day)	19,312	46.83 (34.75)	42.95 (18.22,67.82)	110.04	0, 392.76
HAA5 (µg/day)	17,205	23.32 (21.78)	17.30 (7.83,31.14)	68.48	0, 194.88
TCE (µg/day)	20,428	0.19 (1.31)	0.02 (0.01,0.04)	0.34	0, 36.49
PCE (µg/day)	20,428	0.18 (0.89)	0.02 (0.01,0.03)	0.66	0, 21.37
CWS					
CWS average (2016–2020)					
Arsenic (µg/l)	22,401	0.98 (1.19)	0.48 (0.23,1.31)	3.31	0.01, 15.04
Uranium (µg/l)	19,332	3.40 (3.73)	2.08 (1.21,4.32)	10.87	0.07, 142.48
Gross alpha (pCi/l)	21,121	2.50 (2.41)	1.75 (0.99,3.08)	7.32	0.03, 24.38
Nitrate-N (mg/l)	22,567	1.13 (1.47)	0.31 (0.10,1.76)	4.45	0.02, 7.55
TTHM (µg/l)	18,897	11.67 (9.42)	9.60 (4.95,14.61)	32.02	0.28, 79.80
HAA5 (µg/l)	21,222	23.46 (14.19)	24.31 (12.00,34.21)	47.52	0, 135.52
TCE (µg/l)	22,437	0.09 (0.60)	0.01 (0.01,0.02)	0.16	0, 9.07
PCE (µg/l)	22,437	0.09 (0.41)	0.01 (0.01,0.02)	0.33	0, 5.73

Daily intake was calculated as contaminant concentration (µg/l, pCi/l, mg/l)*Intake Rate (l/day), among participants assigned as CWS users and self-reporting municipal water at questionnaire 6 (2017–2019). CWSs were assigned to CTS participants by residential address at questionnaire 6; average (2016–2020) CWS concentrations among participants assigned as CWS users and self-reporting municipal water at questionnaire 6 are described.

^a^Water intake was calculated from the self-reported number of glasses of home tap water consumed per day. Responses that were recorded as skipped or never were assumed to be 0 glasses of water. Participants were excluded if they had a missing self-reported tap water intake.

^b^Water intake was calculated from the self-reported number of glasses of water, and number of cups of tea and coffee consumed per day (made from home tap water). Participants were excluded if they did not self-report water, tea, or coffee consumption.

Among 27,594 participants with self-reported information about home tap water filtration/treatment, 52% used filtered/treated tap water, 45% did not filter or treat their tap water, and 3% did not know (Table S5). Fifty-four percent of self-reported municipal water users and 47% of domestic well users reported using some type of filtration/treatment. The most common types of treatment specified were refrigerator filters (39%) followed by pitcher filters such as Brita/PUR® (30%); only 15% used reverse osmosis treatment. The use of reverse osmosis was higher among self-reported private well users (27%) compared to municipal water users (15%). Participants that used “other” filtration/treatment methods (16%), reported using distillation, zero water® filters, and boiling water, to name a few examples.

We assessed the agreement between the self-reported and assigned CWS name. Among 23,356 participants with self-reported municipal water, 9834 (40%) provided a water company name. Of those, 9567 participants could be assigned to a CWS based on their address (the other 267 were assigned as domestic well users). The self-reported and assigned CWS name agreed for 8591 participants (90%).

In analyses evaluating temporal changes in 10-year average CWS concentrations for 2000–2009 and 2010–2020 compared to the 1990–1999 average, we observed statistically significant declines in CWS arsenic, uranium, gross alpha, and nitrate, while TTHM concentrations were statistically significantly higher (Table S6). Compared to the 2000–2009 average concentrations, HAA5 concentrations were significantly higher in 2010–2020. No clear temporal trend was observed for TCE and PCE.

## Discussion

We successfully linked CTS participants residing in California to their drinking water source at enrollment through the 6th survey (2017–2019), and characterized long-term exposures to eight regulated water contaminants, leveraging water quality monitoring data for CWS and domestic wells. We observed high consistency between self-reported and assigned water source, and high agreement between self-reported water system name and assigned CWS name, among a subset of participants who completed the 2017–2019 survey. Participant-reported information about drinking water source and CWS name are useful to validate exposure assessments that rely on spatial intersections of geocoded addresses with CWS service area boundaries.

We calculated contaminant intake via tap water based on self-reported information on drinking water source and consumption. Additionally, by assessing the proportion of participants that use tap water filtration or treatment, we can better understand how CWS exposures may differ within a service area, based on point-of-use filtration or treatment. While over half of participants said they drank filtered tap water, only a small percent reported using treatment techniques (e.g., reverse osmosis) that would be likely to remove arsenic, nitrate, and uranium. Additional treatment techniques may have been used that were not captured by the CTS questionnaire. Even among participants who used drinking water treatments or who self-reported ingesting bottled water in 2017–2019 (15%), dermal and inhalation exposure remain potential routes of exposure for some water contaminants like DBPs and VOCs.

Average CWS nitrate exposures were similar comparing CTS participants (median = 0.54 mg/l) to the median nitrate concentration estimated for the population using CWS in the state of California from 2011–2019 (~0.6 mg/l), while median arsenic concentrations were slightly higher in the CTS (1.03 µg/l) compared to statewide estimates (~0.6 µg/l) [58]. CWS nitrate levels in the CTS were slightly lower than previous estimates for the Iowa Women’s Health Study (1.07 mg/l), while TTHM concentrations were higher in our cohort (median = 12.86 µg/l compared to 4.77 µg/l) [36, 59]. CWS arsenic exposures were similar to estimates in the National Health and Nutrition Examination Survey (NHANES, CWS exposures assigned by residential county, median arsenic = 1.35 µg/l), a series of cross-sectional surveys representative of the non-institutionalized general U.S. population [60]. CWS arsenic and uranium (median = 3.48 µg/l) exposures in the CTS were also similar to those in the Multi-Ethnic Study of Atherosclerosis (MESA, exposures assigned by zip code; median arsenic and uranium = 0.35 and 1.14 µg/l, respectively), a prospective cohort of urban, racially and ethnically diverse U.S. adults across six urban centers including Los Angeles [61]. California participants in MESA had CWS arsenic concentrations ranging from 0.35–5.86 µg/l [62]. We are not aware of other U.S. epidemiologic cohorts that have assessed exposure to gross alpha. Differences between studies in the time periods assessed, regions, and amount of monitoring data available, may contribute to the differences observed between our and other studies. Still, similar patterns and magnitudes compared to other cohorts are notable given the geographic and demographic differences in study populations.

We observed differences in drinking water exposures across participant race and ethnicity group and census block group-level SES and urbanicity. Black and Hispanic participants had elevated arsenic, uranium, gross alpha, TCE, and PCE exposures compared to non-Hispanic white participants (Fig. 1). Other race and ethnic groups and multi-racial participants had elevated arsenic, nitrate, TCE, and PCE exposures. This study is consistent with prior findings that CWS arsenic and nitrate concentrations increased per 10% increase in the Latinx population served by CWS in California (2011–2019) [58]. CWS arsenic concentrations (2006–2011) were also higher among Hispanic, Black, and Chinese-American participants compared to Non-Hispanic white participants in California in MESA [61]. Native American participants had elevated uranium and gross alpha exposures, which is consistent with the legacy of uranium mining and milling on or near tribal areas in the American west [63, 64]. Arsenic, uranium, gross alpha, and nitrate exposures were lower in upper SES and metropolitan areas, while DBP exposures were higher. Previous studies have documented disparities in drinking water metal/metalloid, nitrate, and other regulated contaminants in the U.S. by region, SES, race and ethnicity, and rurality/urbanicity [10, 41, 42, 65].

Prior evidence on differences in HAA5, TTHM, TCE, and PCE exposures across subpopulations is limited; our study underscores the need for future evaluations of DBP and VOC exposures in drinking water. As expected, TTHM and HAA5 concentrations were higher among CWS users compared to domestic well users; CWS users would be more likely to have DBP exposures than domestic well users due to the regular use of chlorination and other disinfection processes for CWS, compared to less frequent disinfection of domestic wells. The groundwater quality data used to estimate exposures to areas served by domestic wells included some data from CWS wells, which may overestimate TTHM and HAA5 concentrations for domestic well users. Though TCE and PCE concentrations were higher among CWS users compared to domestic well users, detections of TCE and PCE in CWS were reported for only about 35% of years (population-weighted average) from 1990–2020 (Table S2).

### Strengths and limitations

Strengths of this study include our use of long-term water quality monitoring data for multiple regulated contaminants with extensive geographic coverage across California. Epidemiologic analyses of drinking water exposures have previously been limited by the inability to link water quality data to epidemiologic cohorts. Based on enrollment addresses, we were able to match all CTS participants living in California to a CWS or domestic well exposure estimate. We assessed assignment accuracy and potential exposure misclassification at follow-up (Q6, 2017–2019). Bottled water use was the primary reason for potential exposure misclassification of the drinking water source; however, additional routes of exposure to water contaminants, such as dermal exposure to DBPs, remain relevant even to participants who did not report drinking their home tap water [66]. Almost all of the self-reported municipal water users were correctly found to live within CWS boundaries (22,697 of 23,356; 97%), and most self-reported private well users were correctly found to live outside of CWS boundaries (1345 of 1927; 70%). However, some participants who were spatially assigned as domestic well users self-reported drinking municipal water (26%); this is likely explained by CWS service boundaries lacking sufficient granularity to pinpoint households within the service area that relied on a private well. This information may be used to inform and improve future updates to the shapefiles of CWS service boundaries. With high agreement between assigned vs. self-reported water system name (90%), our findings suggest that exposure misclassification of CWS source is not likely to be large. We did not have information at enrollment on home drinking water consumption; however, per capita consumption of bottled water has generally increased in the U.S. from 1999–2022 [67]. Data from NHANES suggests that prevalence of home tap water consumption (vs. bottled water), was consistent for U.S. adults between 2007–2016 [68]. Our findings of median tap water intake (1.42 l of water, 2.01 l of water, tea, and coffee) were comparable to previous estimates of tap water intake for women in NHANES [69, 70]. Few U.S. cohort studies have quantified tap water contaminant exposures using self-reported information on home tap water consumption, though this represents an important component to estimating drinking water dose and can enable more precise evaluation of toxicant dose-response relationships [71].

Limited CWS data were available for uranium and HAA5. For uranium, CWS were permitted by Section 64442(f) of Title 22 of the California Code of Regulations to substitute gross alpha activity measurements for uranium measurements, if the gross alpha concentration did not exceed 5 pCi/l [44]. As such, CWS (particularly small (>500–3300 people served) and very small (≤500 people) systems with gross alpha <5 pCi/l had more missing uranium measurements. Future studies may consider the use of gross alpha and other co-contaminants, and hydrological and geochemical characteristics of the water source, to estimate uranium when measurement data are sparse. For HAA5, the data were available for a limited number of CWS prior to 2003 (CWS *N* = 14). In the absence of historical monitoring data, it may be reasonable to use recent HAA5 data to estimate past exposures if water sources and treatment methods did not change. When earlier CWS data are available, future studies may also evaluate whether HAA5 levels were stable over time when treatment methods and water sources did not change. Additional limitations include our inability to evaluate drinking water sources and exposures outside of the home, such as places of employment and recreation. We did not consider the residential history in this analysis, and linked participants to their corresponding water source based on their address at enrollment and at the 6th survey. Future analyses that use the complete residential history should provide improved exposure estimates that allow for lagging of exposures as appropriate for the health outcome being studied.

### Conclusions

In this assessment of drinking water exposures for the California Teachers Study, we generated estimates of long-term exposure for study participants that will be useful for epidemiologic studies of chronic disease outcomes. This study demonstrated that linkage of epidemiologic study populations’ address data to CWS boundaries represents a reasonable approach that is not likely to introduce substantial exposure misclassification. We observed heterogeneity in CWS exposures by participant race and ethnicity, and neighborhood SES and urbanicity. Identification of differential exposure to water contaminants is critical to the development of effective public health interventions that reduce drinking water exposures, exposure disparities, and associated health risks [72].

## Supplementary information

Supplemental Tables and Figures

## Data Availability

All of the data associated with this publication and in the California Teachers Study are available for research use. The California Teachers Study welcomes all such inquiries and encourages individuals to visit https://calteachersstudy.my.site.com/for-researchers. Investigators interested in analyzing OEHHA data may contact OEHHA and access the CalEnviroScreen Data Dashboard. The statistical code for analysis is available upon reasonable request, please contact MS at maya.spaur@nih.gov.
