# Supplementary material for: Drinking water source and exposure to regulated water contaminants in the California Teachers Study cohort
Source: J Expo Sci Environ Epidemiol. 2024 Jul 13;35(3):454–65. doi: 10.1038/s41370-024-00703-9 (PMC12069093; doi:10.1038/s41370-024-00703-9)
Supplement: Supplementary file 1 — Supplemental Tables and Figures [file 41370_2024_703_MOESM1_ESM.docx]

**Supplemental Tables and Figures**

| **Page (s)** | **Table** | **Title** |
| --- | --- | --- |
| 2 | S1 | Detection limits for the purposes of reporting (DLR), and upper bound concentrations used for imputing concentrations <DLR. |
| 3 | S2 | Number of community water systems (CWS) with annual average measurements and distributions of the percent of years with detectable concentrations (not imputed) for water quality monitoring data (1990-2020). |
| 4 | S3 | Urbanization category definitions. |
| 5 | S4 | Distributions of long-term (1990-2015) average concentrations of community water system (CWS) exposures, and percent of years the annual average concentration was at or above the maximum contaminant level (MCL) and half of the MCL, among participants with a residential duration at their enrollment address > 20 years. |
| 6 | S5 | Home tap water filtration responses at the 6th follow-up (2017-2019) by self-reported water source (N=27,594), and the type of water filtration method used (N=7,774) for participants who completed the paper version of the questionnaire. |
| 7 | S6 | Median concentrations (IQRs) and adjusted geometric mean ratios (GMRs, 95% CI) of 10-year average concentrations of community water system (CWS) arsenic (As), uranium (U), gross alpha, nitrate-nitrogen, five haloacetic acids (HAA5), total trihalomethanes (TTHM), trichloroethylene (TCE) and tetrachloroethylene (PCE), over the 1990-2020 period. |
| **Page (s)** | **Figure** | **Title** |
| 8 | S1 | Timeline of the California Teachers Study. |
| 9 | S2 | Map of community water systems (CWS) serving California Teachers Study participants in California. |
| 10 | S3 | Spearman correlation coefficients of long-term average (1990-2015) community water system (CWS) arsenic (As), uranium (U), gross alpha, nitrate-nitrogen (N), five haloacetic acids (HAA5), total trihalomethanes (TTHM), trichloroethylene (TCE), and tetrachloroethylene (PCE) concentrations among California Teachers Study (CTS) participants. |
| 11 | S4 | Number (N) of community water systems (CWS) reporting annual water quality data by year and contaminant, linked to California Teachers Study participants based on address at enrollment. |
| 12-13 | S5 | Distribution of drinking water contaminant concentrations (long-term average, 1990-2015) by participant race and ethnicity. |
| 14-15 | S6 | Distribution of drinking water contaminant concentrations (long-term average, 1990-2015) by census block group-level socioeconomic status (SES) quartile. |
| 16-18 | S7 | Unadjusted percent (%) change (95% CI) of long-term (1990-2015) average concentrations of community water system (CWS) exposures by census block group-level socioeconomic status quartile (SES) and urbanicity, and participant race and ethnicity (N=114,183 participants with race/ethnicity, SES, and urbanicity information). |

**Table S1. Detection limits for the purposes of reporting (DLR), and upper bound concentrations used for imputing concentrations <DLR^1^.**

| **Contaminant** | **Unit** | **DLR** | **Upper bound** |
| --- | --- | --- | --- |
| Arsenic | µg/L | 2 | 1 (prior to 2008); 0.5 (2008 onwards) |
| Uranium | pCi/L | 1 | 0.5 |
| Gross Alpha | pCi/L | 3 | 1 |
| Nitrate as N | mg/L | 0.4 | 0.1 |
| Sum of 5 haloacetic Acids (HAA5) | µg/L | 0.0 (None) | 2 |
| Total Trihalomethanes (TTHM) | µg/L | 0.0 (None) | 0.5 |
| Trichloroethylene (TCE) | µg/L | 0.5 | 0.1 |
| Tetrachloroethylene (PCE) | µg/L | 0.5 | 0.1 |

^1^When samples had a concentration of 0 or were reported as “below the DLR”, we imputed concentrations using Tobit regression based on a log-normal distribution for the existing measurement data. The upper bound for imputation was derived from the median of reported concentrations below the DLR and the lower bound was zero.

**Table S2. Number of community water systems (CWS) with annual average measurements and distributions of the percent of years with detectable concentrations (not imputed) for water quality monitoring data (1990-2020)^1^.** CWS data were linked by enrollment address.

|  | **N CWS with at least one year of measurement data (including imputed values)^2^** | **N CWS with > 1 year of detectable data^3^** | **Median (IQR) % years of detectable data^4^** | **Mean (SD) % years of detectable data^5^** | **Population-weighted Mean (SD) % years of detectable data^6^** |
| --- | --- | --- | --- | --- | --- |
| Arsenic | 1227 | 1163 | 54 (14, 88) | 51 (37) | 64 |
| Uranium | 901 | 818 | 100 (68, 100) | 81 (32) | 80 |
| GA | 1224 | 1149 | 90 (73, 100) | 83 (21) | 86 |
| Nitrate | 1232 | 1130 | 100 (60, 100) | 77 (39) | 83 |
| HAA5 | 988 | 978 | 97 (50, 100) | 73 (36) | 88 |
| TTHM | 1239 | 1229 | 54 (27, 77) | 52 (32) | 70 |
| TCE | 1228 | 1156 | 0 (0, 0) | 8 (23) | 28 |
| PCE | 1228 | 1156 | 0 (0, 0) | 10 (26) | 35 |

^1^Contaminants are abbreviated as follows: gross alpha (GA), nitrate-nitrogen (Nitrate), five haloacetic acids (HAA5), total trihalomethanes (TTHM), trichloroethylene (TCE) and tetrachloroethylene (PCE). Samples that were above the detection limits for the purposes of reporting (DLR) were not imputed; samples that were reported as equal to, half of, or below the DLR were imputed**.** The upper bound for imputation was contaminant specific and was derived from the median of reported concentrations below the DLR; the lower bound was zero.

^2^N CWS = number of CWS with at least one year of measurement data (including imputed and detectable [not imputed] data).

^3^N CWS = number of CWS with at least one year of measurement data that was detectable and not imputed (sample measurement was above the DLR and did not equal zero).

^4^Median (IQR) = median (interquartile range, IQR) of the % years of detectable data, calculated as the total N of years that CWS had detectable measurement data [not imputed] / total N of years of data [imputed and not imputed].

^5^Mean (SD) = mean (standard deviation) of the % years of detectable data, calculated as the total N of years that CWS had detectable measurement data [not imputed] / total N of years of data [imputed and not imputed].

^6^Mean of the % years of detectable data, calculated as the total N of years that CWS had detectable measurement data [not imputed] / total N of years of data [imputed and not imputed], weighted by the population served. Population served information was provided by OEHHA based on 2019-2020 data collected from the Safe Drinking Water Information System (SDWIS).

**Table S3. Urbanization category definitions.** Urbanization codes were classified using census block group-level population data from the 1990 Census by a multi-step process described in the footnote.

|  | **Urbanized Area^1^** | **Census Place^2^** | **Block^3^** | **Block Group^4^** |
| --- | --- | --- | --- | --- |
| **Description** | **Population size** | **Population size** | **Population size** | **Population density** |
| Metropolitan Urban | >1,000,000 |  |  | >75^th^ % percentile |
| Metropolitan Suburban | >1,000,000 |  |  | <75^th^ % percentile |
| City | <1,000,000 | >50,000 |  |  |
| Town |  | <50,000 |  | >25^th^ % percentile |
| Rural |  | <50,000 | >0 | <25^th^ % percentile |

^1^First, census-defined Urbanized Areas were assigned to census Blocks; metropolitan urban areas were defined as having a population >1,000,000 and non-metropolitan city urban areas were defined as population <1,000,000.

^2^Population information from census Places were used to further describe census Blocks as a city (population >50,000) or town (population <50,000).

^3^Census Blocks that were not assigned to the before-mentioned categories were classified as rural.

^4^Census Blocks were aggregated to census Block Groups per the following process: Block Groups consisting of Blocks with the same urban code were categorized as that code; Block Groups consisting of Blocks with different urban codes were assigned an urban code based on the population distribution, such that if any urban code accounted for >50% of the population then that urban code was assigned to the Block Group. For Block Groups that remained unassigned, the highest-ranking urban code (that accounted for >25% of the population) was assigned. Additional adjustments were made to incorporate population density information: Block Groups coded as a metropolitan urban area with a population density in the top quartile retained their metropolitan urban code, and the remaining metropolitan Block Groups (in the lower three quartiles of population density) were re-categorized as metropolitan suburban. Block Groups that were categorized as towns were re-categorized to rural if their population density was in the lowest quartile.

**Table S4. Distributions of long-term (1990-2015) average concentrations of community water system (CWS) exposures^1^, and percent of years the annual average concentration was at or above the maximum contaminant level (MCL) and half of the MCL^2^, among participants with a residential duration at their enrollment address > 20 years.**

|  | **Average concentration** | | | | | |
| --- | --- | --- | --- | --- | --- | --- |
|  | **N Participants** | **N CWS** | **Mean (SD)** | **Median (25th, 75th)** | **95th %** | **Range^3^** |
| Arsenic (µg/L) | 60742 | 970 | 1.40 (1.61) | 1.03 (0.51,1.67) | 3.78 | 0.01, 34.44 |
| Uranium (µg/L) | 58972 | 718 | 4.40 (4.08) | 3.42 (1.01,6.17) | 12.63 | 0.03, 88.8 |
| Gross alpha (pCi/L) | 60695 | 961 | 2.74 (1.94) | 2.21 (1.28,3.67) | 6.16 | 0.2, 55.28 |
| Nitrate-N (mg/L) | 60754 | 975 | 1.29 (1.53) | 0.50 (0.20,1.97) | 4.95 | 0.02, 12.90 |
| HAA5 (µg/L) | 52407 | 729 | 10.43 (8.63) | 9.20 (3.00,14.87) | 29.71 | 0.13, 115.18 |
| TTHM (µg/L) | 60101 | 975 | 14.86 (11.91) | 12.86 (5.01,22.26) | 36.51 | 0, 105.45 |
| TCE (µg/L) | 60741 | 970 | 0.28 (1.32) | 0.01 (0.01,0.17) | 0.74 | 0, 32.89 |
| PCE (µg/L) | 60741 | 970 | 0.24 (1.49) | 0.02 (0.01,0.10) | 0.66 | 0, 25.03 |
|  | **% of years > 1/2 MCL** | | | **% of years > MCL** | | |
|  | **N Participants** | **N CWS** | **Mean (SD)** | **N Participants** | **N CWS** | **Mean (SD)** |
| Arsenic | 19345 | 302 | 4 (13) | 6326 | 141 | 1 (6) |
| Uranium | 15715 | 138 | 5 (12) | 2647 | 40 | 0 (3) |
| Gross alpha | 20348 | 237 | 5 (13) | 6833 | 69 | 1 (2) |
| Nitrate-N | 12983 | 226 | 5 (16) | 1444 | 36 | 0 (1) |
| HAA5 | 7781 | 117 | 5 (16) | 310 | 21 | 0 (1) |
| TTHM | 37597 | 324 | 13 (17) | 2328 | 51 | 0 (1) |
| TCE | 8038 | 46 | 2 (8) | 2594 | 18 | 1 (6) |
| PCE | 7979 | 65 | 2 (8) | 3703 | 35 | 1 (4) |

^1^Community water system arsenic, uranium, gross alpha, nitrate-nitrogen (N), five haloacetic acids (HAA5), total trihalomethanes (TTHM), trichloroethylene (TCE) and tetrachloroethylene (PCE) concentrations were assigned to California Teachers Study (CTS) participants by enrollment address.

^2^The MCLs are as follows: arsenic (10 µg/L), uranium (30 μg/L), gross alpha (15 pCi/L, not including radon and uranium), nitrate-N (10 mg/L), TTHM (80 μg/L), HAA5 (60 μg/L), TCE (5 μg/L), and PCE (5 μg/L). Measurement data below the detection limit for the purposes of reporting (DLR) were imputed using Tobit regression. % of years > ½ MCL = total number of years that the annual average concentration was > ½ MCL / total number of years of measurement data per CWS. % of years > MCL = total number of years that the annual average concentration was > MCL / total number of years of measurement data per CWS. N participants and N CWS indicate the N of participants and N CWS that had at least one annual average concentration > ½ MCL or > MCL.

^3^Range = minimum, maximum.

**Table S5. Home tap water filtration responses at the 6^th^ follow-up (2017-2019) by self-reported water source (N=27,594), and the type of water filtration method used (N=7,774) for participants who completed the paper version of the questionnaire.**

| **Filtration used, N (%)** | **Overall**  **N=27594** | **Self-reported municipal water**  **N=22378** | **Self-reported private well**  **N=1829** |
| --- | --- | --- | --- |
| **Yes** | 14483 (52) | 12092 (54) | 869 (47) |
| **No** | 12412 (45) | 9760 (44) | 932 (51) |
| **Don’t Know** | 699 (3) | 526 (2) | 28 (2) |
| **Filtration method used,**  ***N (%) among paper participants*** | **N=7774^5^** | **Self-reported municipal water**  **N=5841** | **Self-reported private well**  **N=422** |
| Brita or PUR^1^ | 2300 (30) | 1779 (30) | 87 (20) |
| Refrigerator^2^ | 3029 (39) | 2365 (40) | 83 (20) |
| Reverse osmosis^3^ | 1161 (15) | 832 (15) | 113 (27) |
| Other^4^ | 1284 (16) | 865 (15) | 139 (33) |

^1^Questionnaire response was: “Yes, using a filter like Brita or PUR in a pitcher or on a faucet.”

^2^Questionnaire response was: “Yes, filtered using water from my refrigerator.”

^3^Questionnaire response was: “Yes, using reverse osmosis (under sink or whole house).”

^4^Questionnaire response was: “Yes, filtered with another type of filter (under sink or whole house” or “Yes, other.”

^5^Of the 14,509 participants who answered yes to home tap water filtration overall, participants who had completed the mailed version of the questionnaire were able to provide more detailed information on the method of filtration used (N=7,774). No detailed information was asked of participants who filled out the web version and answered “Yes” to home tap water filtration (N=6,735).

**Table S6. Median concentrations (IQRs) and adjusted geometric mean ratios (GMRs, 95% CI)^1^ of 10-year average concentrations of community water system (CWS) arsenic, uranium, gross alpha, nitrate-nitrogen (N), five haloacetic acids (HAA5), total trihalomethanes (TTHM), trichloroethylene (TCE) and tetrachloroethylene (PCE), over the 1990-2020 period.** Restricted to CWS with an average concentration available for all three 10-year periods. HAA5 analyses were restricted to CWS with an average concentration for 1990-2009 and 2010-2020.

|  | **1990-1999** | **2000-2009** | **2010-2020** | **P value** |
| --- | --- | --- | --- | --- |
| **Arsenic, N CWS = 975**^2^ |  |  |  |  |
| Concentration, Median (IQR) | 1.00 (0.51, 2.30) | 0.86 (0.42, 2.23) | 0.72 (0.25, 1.95) |  |
| GMR (95% CI) | 1.0 (ref) | 0.93 (0.84, 1.04) | 0.68 (0.62, 0.76) | <0.001 |
| **Uranium, N CWS = 486**^2^ |  |  |  |  |
| Concentration | 4.76 (2.92, 7.93) | 3.84 (1.87, 7.57) | 3.63 (1.72, 6.96) |  |
| GMR (95% CI) | 1.0 (ref) | 0.81 (0.71, 0.92) | 0.73 (0.65, 0.83) | <0.001 |
| **Gross alpha, N CWS = 946**^2^ |  |  |  |  |
| Concentration, Median (IQR) | 1.85 (1.11, 3.21) | 1.51 (0.79, 2.98) | 1.67 (0.74, 3.43) |  |
| GMR (95% CI) | 1.0 (ref) | 0.85 (0.78, 0.93) | 0.88 (0.81, 0.96) | <0.001 |
| **Nitrate-N, N CWS = 984**^2^ |  |  |  |  |
| Concentration, Median (IQR) | 0.88 (0.29, 2.24) | 0.83 (0.23, 2.17) | 0.80 (0.14, 2.33) |  |
| GMR (95% CI) | 1.0 (ref) | 0.93 (0.82, 1.05) | 0.83 (0.74, 0.94) | <0.001 |
| **TTHM, N CWS = 932**^2^ |  |  |  |  |
| Concentration, Median (IQR) | 0.26 (0.09,3.93) | 1.02 (0.16, 9.73) | 9.41 (1.73, 27.49) |  |
| GMR (95% CI) | 1.0 (ref) | 2.64 (2.21, 3.15) | 11.66 (9.77, 13.92) | <0.001 |
| **TCE, N CWS = 938**^2^ |  |  |  |  |
| Concentration, Median (IQR) | 0.01 (0.00, 0.02) | 0.01 (0.01, 0.02) | 0.01 (0.01, 0.01) |  |
| GMR (95% CI) | 1.0 (ref) | 0.94 (0.80, 1.12) | 0.96 (0.81, 1.13) | 0.61 |
| **PCE, N CWS = 938**^2^ |  |  |  |  |
| Concentration, Median (IQR) | 0.01 (0.00,0.02) | 0.01 (0.01, 0.02) | 0.01 (0.01, 0.02) |  |
| GMR (95% CI) | 1.0 (ref) | 1.20 (0.99, 1.45) | 1.12 (0.93, 1.35) | 0.24 |
| **HAA5, N CWS = 448**^3^ |  | **2000-2009** | **2010-2020** | **P value** |
| Concentration, Median (IQR) |  | 4.98 (1.34, 17.00) | 7.82 (2.55, 18.92) |  |
| GMR (95% CI) |  | 1.0 (ref) | 1.39 (1.22, 1.59) | <0.001 |

^1^Generalized linear models were used to estimate GMRs of 10-year average CWS concentrations (1990-1999 average concentration as reference), with adjustment for water source type (groundwater, groundwater under the influence of surface water, surface water) and population served (very small (<500 people), small (>500-3,300 people), medium (>3,300-10,000 people), large (>10,000-<1,000,000 people), and very large (>1,000,000 people)). Arsenic concentrations >1000 were excluded from analyses.

^2^N CWS with an average concentration available for all 10-year intervals.

^3^N CWS with an average HAA5 concentration available for 2000-2009 and 2010-2020. Only 5 CWS had an average HAA5 concentration available for 1990-1999.

**Figure S1. Timeline of the California Teachers Study.** Water quality monitoring data for community water systems (CWS) were available from 1990-2020 for arsenic, uranium, gross alpha, nitrate-nitrogen (nitrate-N), total trihalomethanes (TTHM), trichloroethylene (TCE), and tetrachloroethylene (PCE) and from 1992-2020 for five haloacetic acids (HAA5). **S**ource: <https://www.calteachersstudy.org/cts-data>.


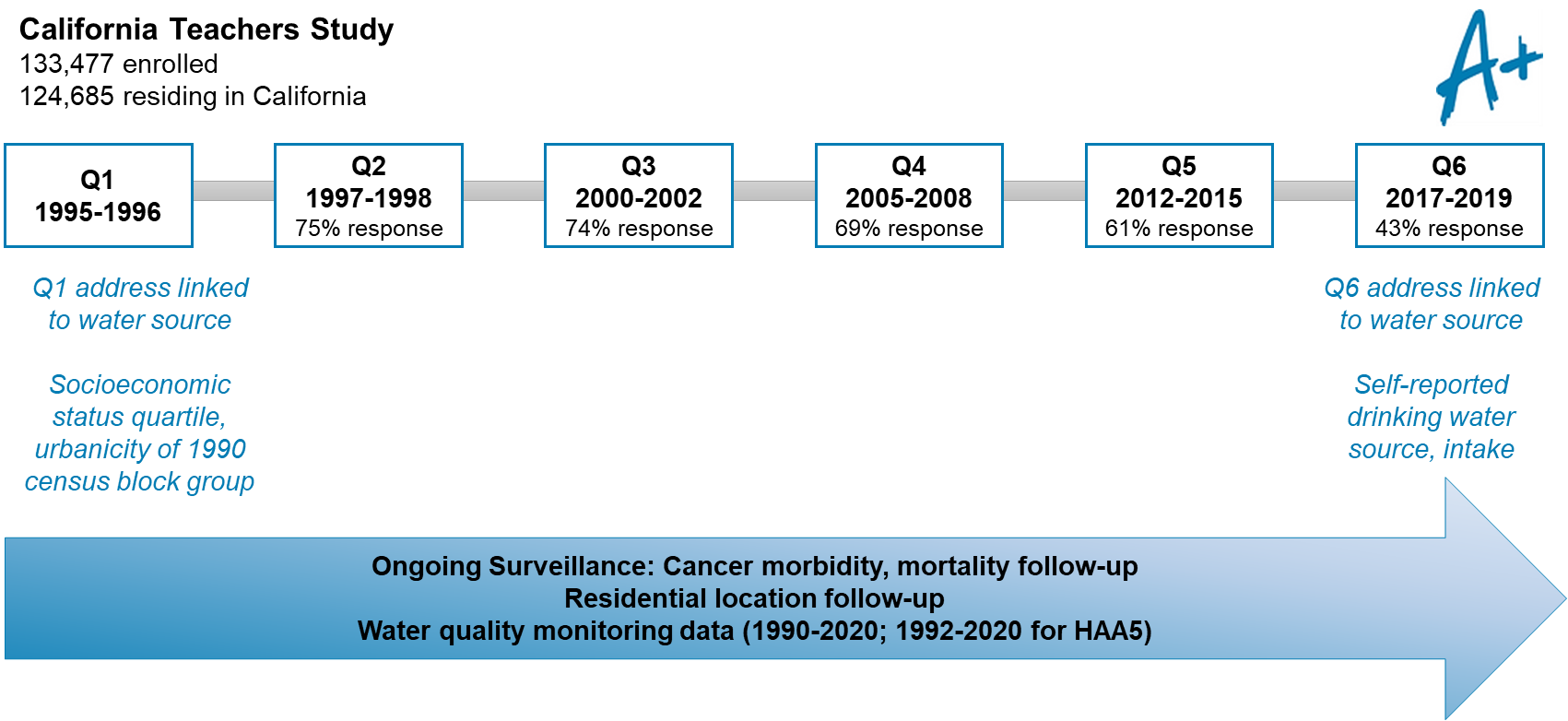


**Figure S2. Map of community water systems (CWS) serving California Teachers Study participants in California.** CWS that serve > 5 participants are shown.

**
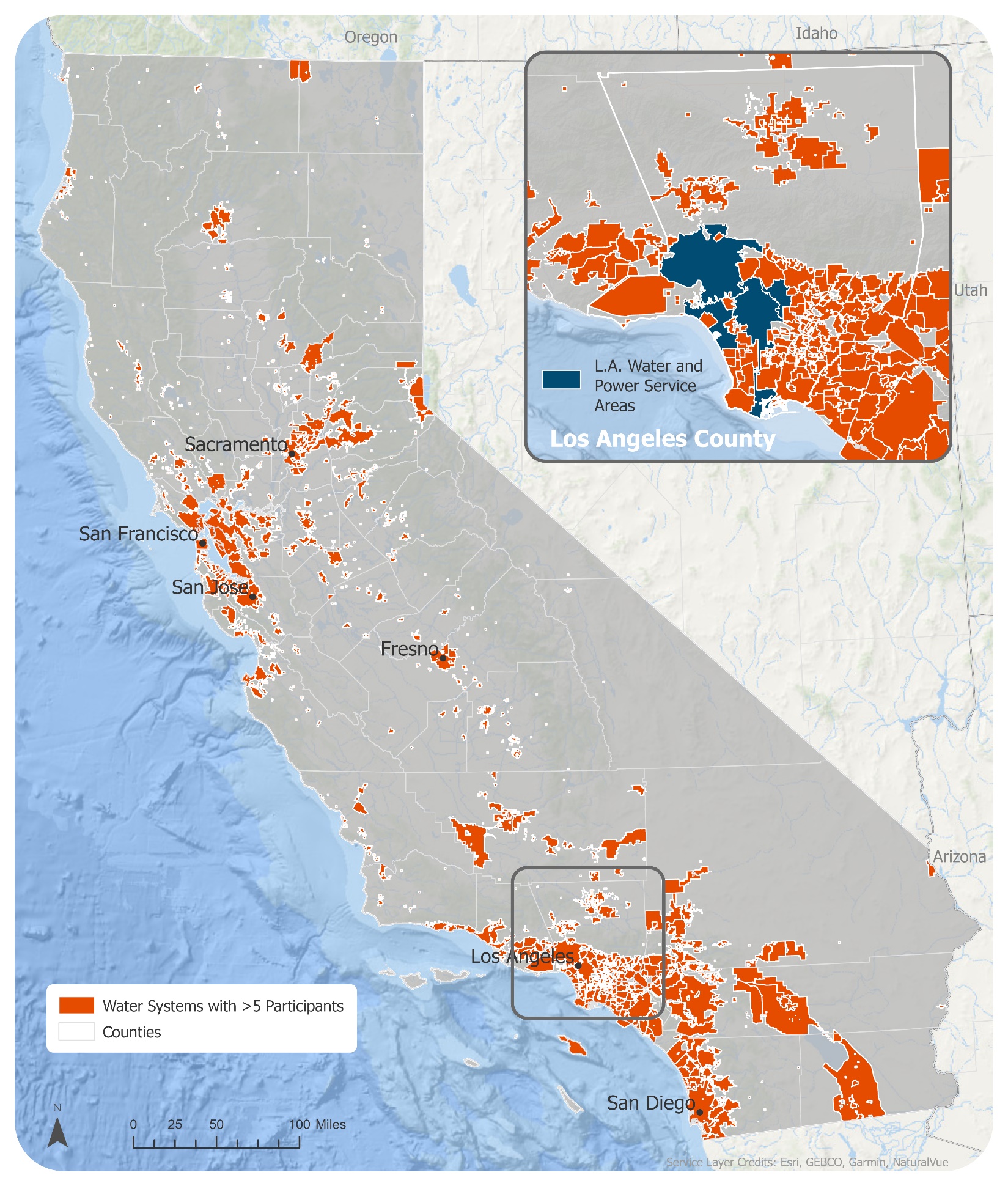
**

**Figure S3. Spearman correlation coefficients of long-term average (1990-2015) community water system (CWS) arsenic, uranium, gross alpha, nitrate-nitrogen (N), five haloacetic acids (HAA5), total trihalomethanes (TTHM), trichloroethylene (TCE), and tetrachloroethylene (PCE) concentrations among California Teachers Study (CTS) participants.** Positive correlations are shown in blue and negative correlations are shown in red.


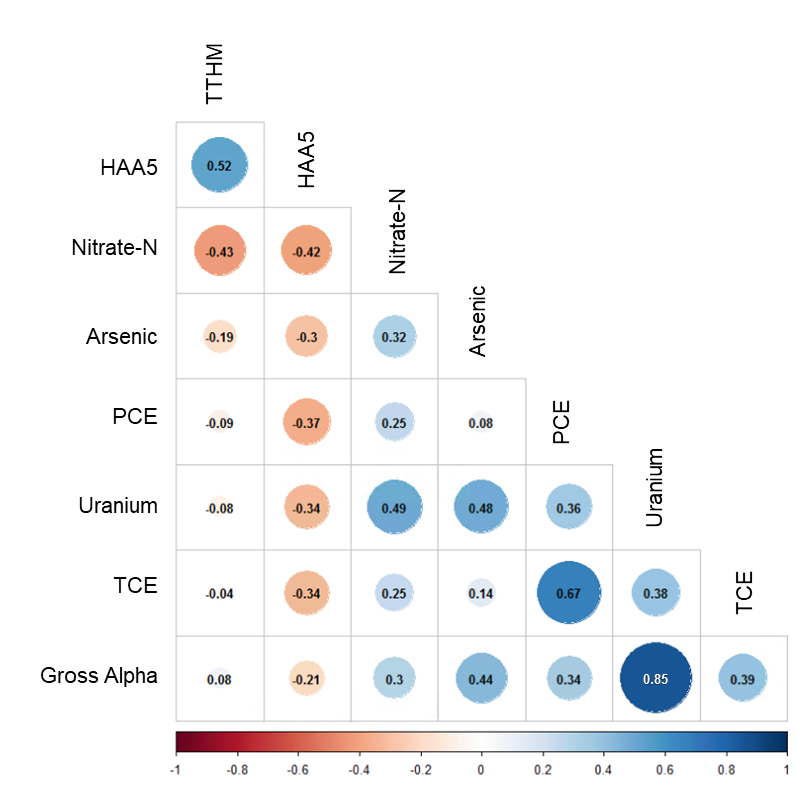


**Figure S4. Number (N) of community water systems (CWS) reporting annual water quality data by year and contaminant, linked to California Teachers Study participants based on address at enrollment.** Contaminants are arsenic, uranium, gross alpha, nitrate-nitrogen (N), five haloacetic acids (HAA5), total trihalomethanes (TTHM), trichloroethylene (TCE), and tetrachloroethylene (PCE).


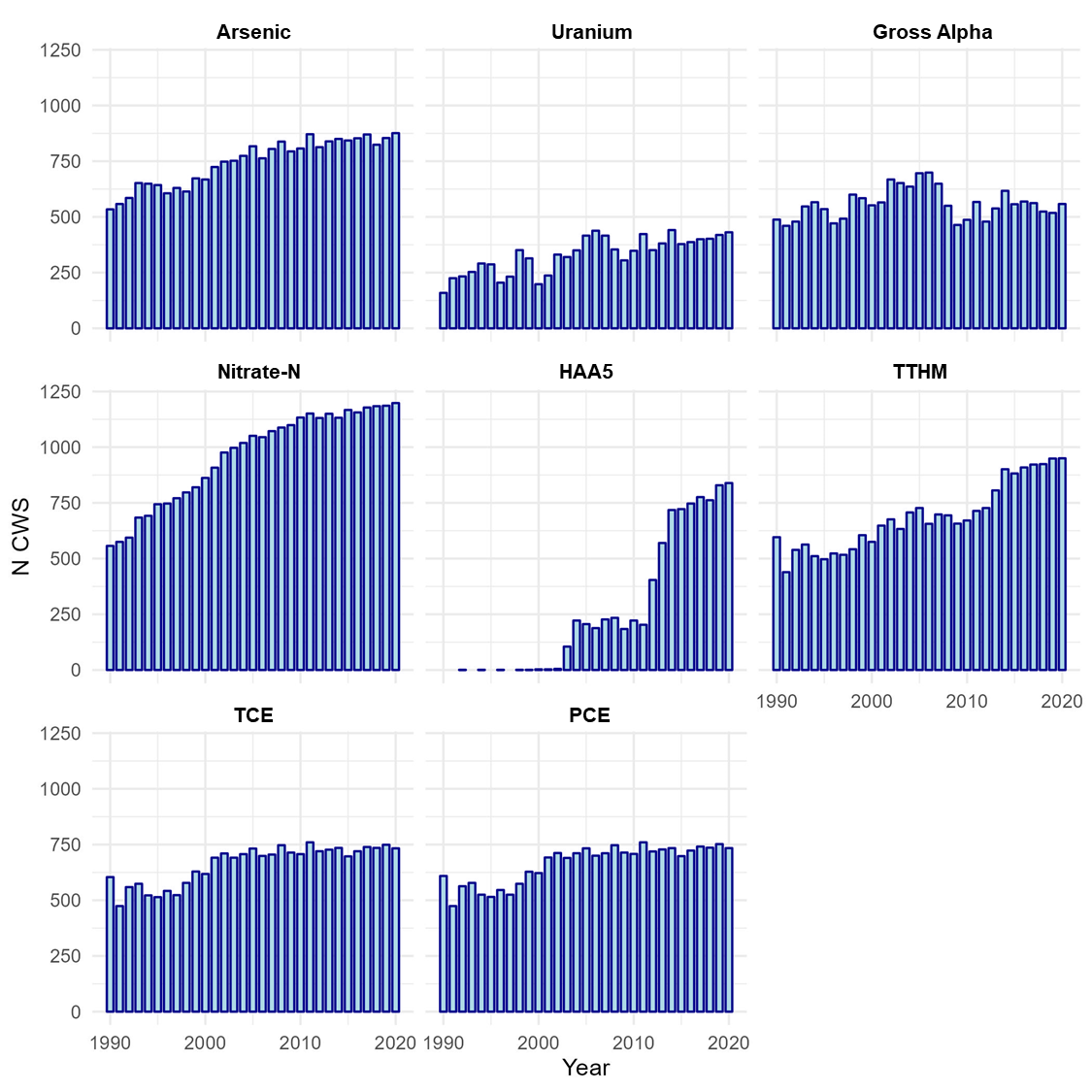


**Figure S5. Distribution of drinking water contaminant concentrations (long-term average, 1990-2015) by participant race and ethnicity.** Red vertical line indicates the current U.S. EPA MCL for each contaminant. Contaminants are arsenic (As), uranium (U), gross alpha (GA, nitrate-nitrogen (NitN), five haloacetic acids (HAA5), total trihalomethanes (TTHM), trichloroethylene (TCE), and tetrachloroethylene (PCE).

**
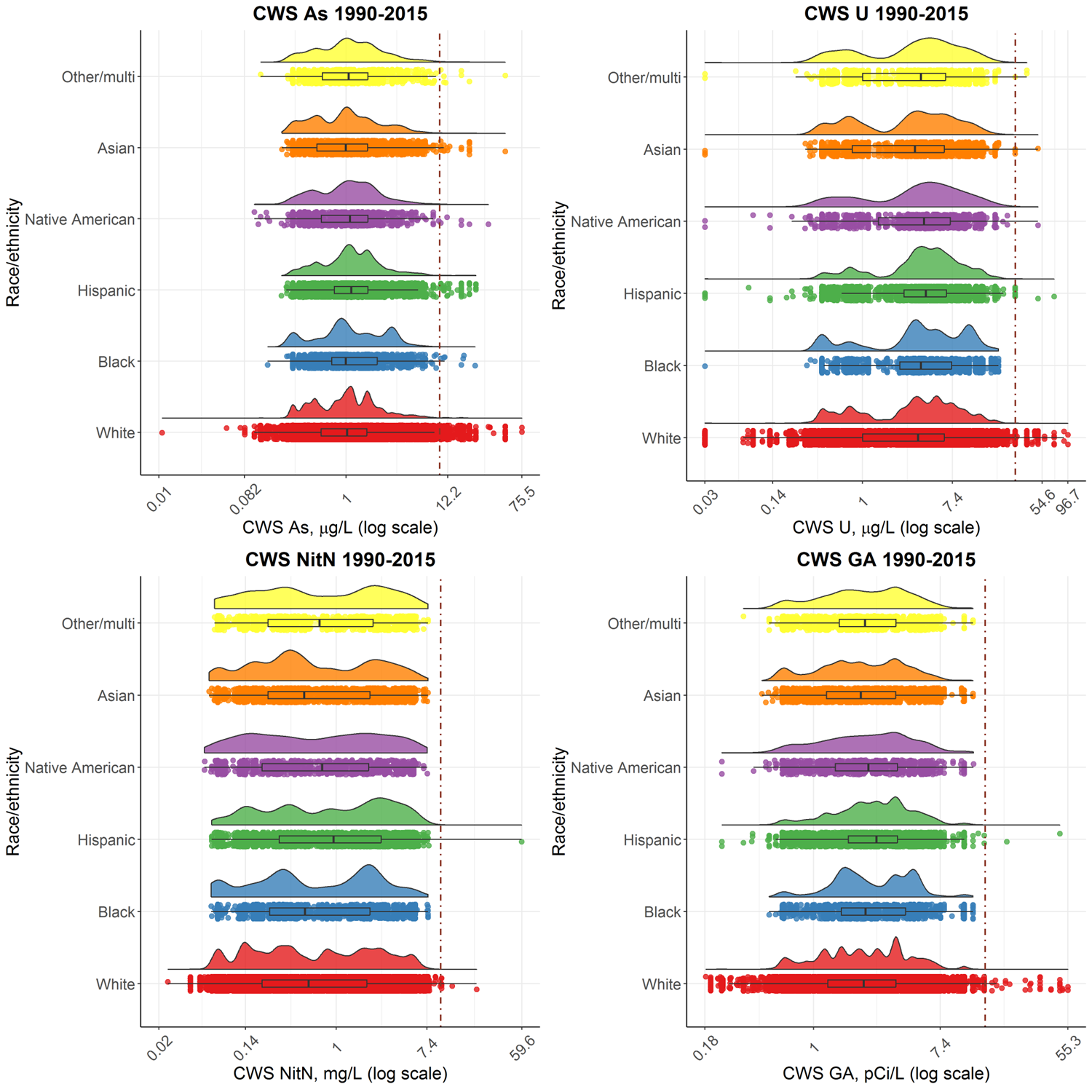
**

**
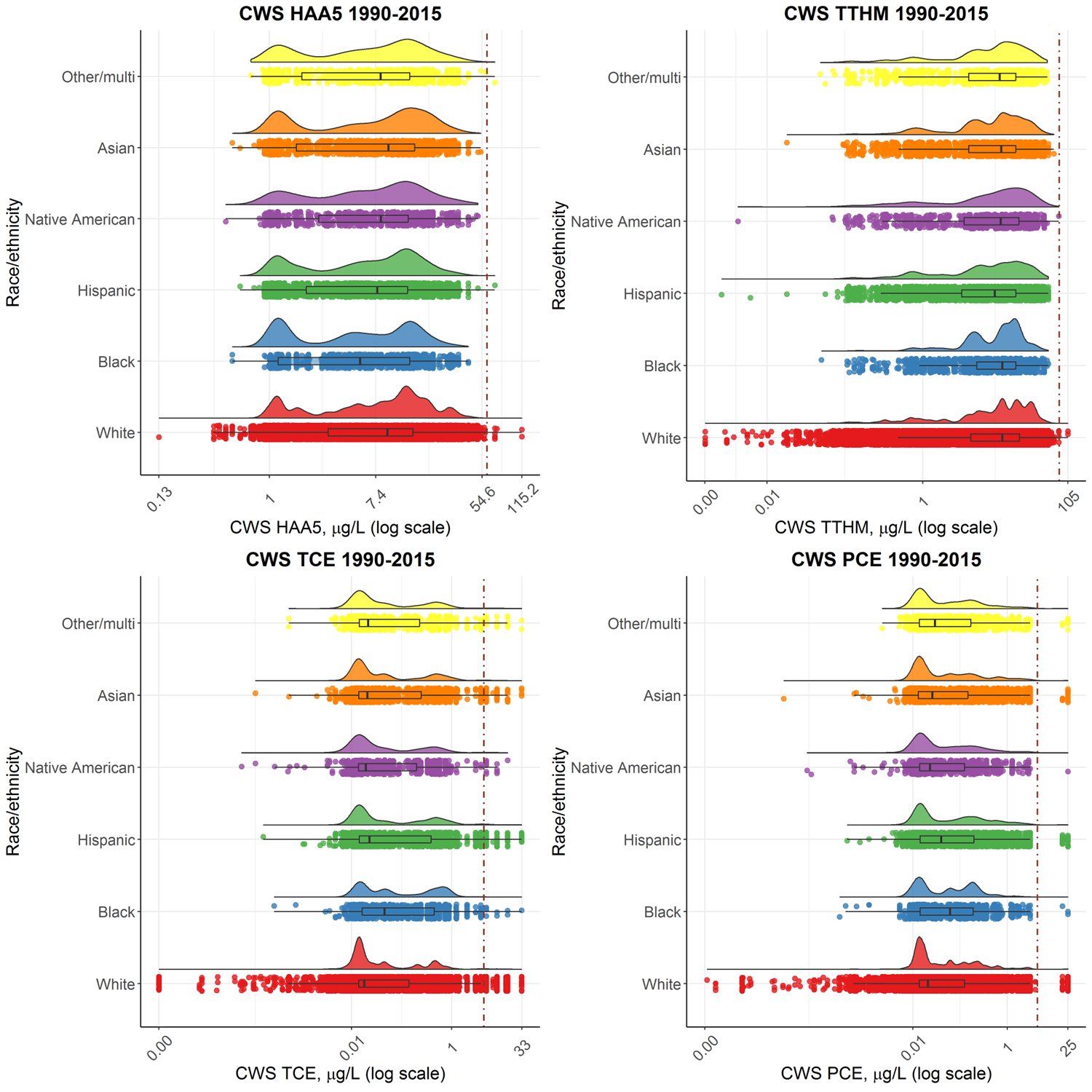
**

**Figure S6. Distribution of drinking water contaminant concentrations (long-term average, 1990-2015) by census block group-level socioeconomic status (SES) quartile.** Red vertical line indicates the current U.S. EPA MCL for each contaminant. Contaminants are arsenic (As), uranium (U), gross alpha (GA, nitrate-nitrogen (NitN), five haloacetic acids (HAA5), total trihalomethanes (TTHM), trichloroethylene (TCE), and tetrachloroethylene (PCE). SES metric was created incorporating three 1990 census block group variables (occupation, education, and income) (Hurley et al. 2005).


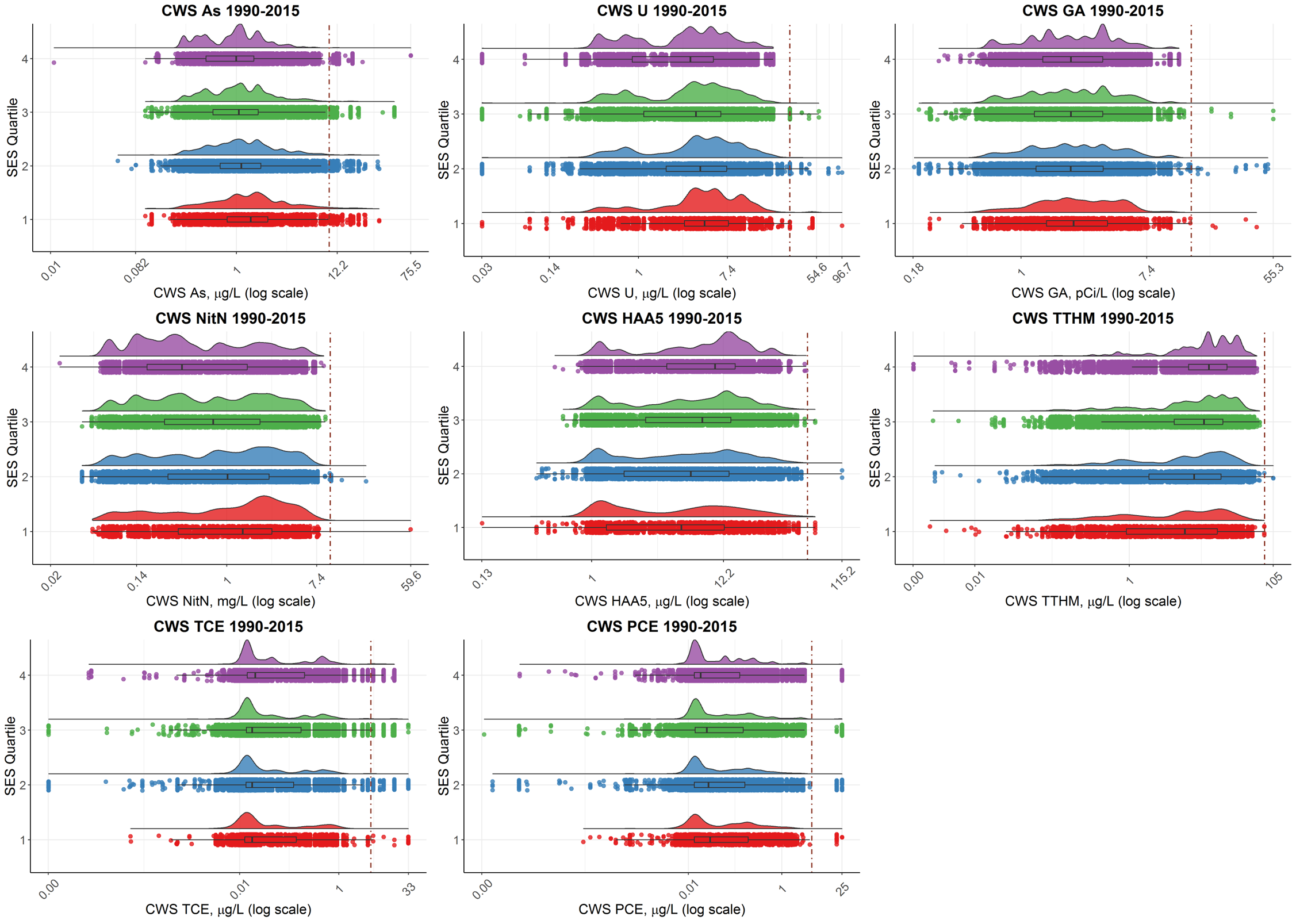


**Figure S7. Unadjusted percent (%) change (95% CI)^1^ of long-term (1990-2015) average concentrations of community water system (CWS) exposures^2^ by census block group-level socioeconomic status quartile (SES)^3^ and urbanicity^4^, and participant race and ethnicity (N=114,183 participants with race and ethnicity, SES, and urbanicity information).** Reference groups are in black, non-reference groups are in blue. Shapes represent each sociodemographic group category (square = urbanicity, triangle = SES, circle = race/ethnicity).


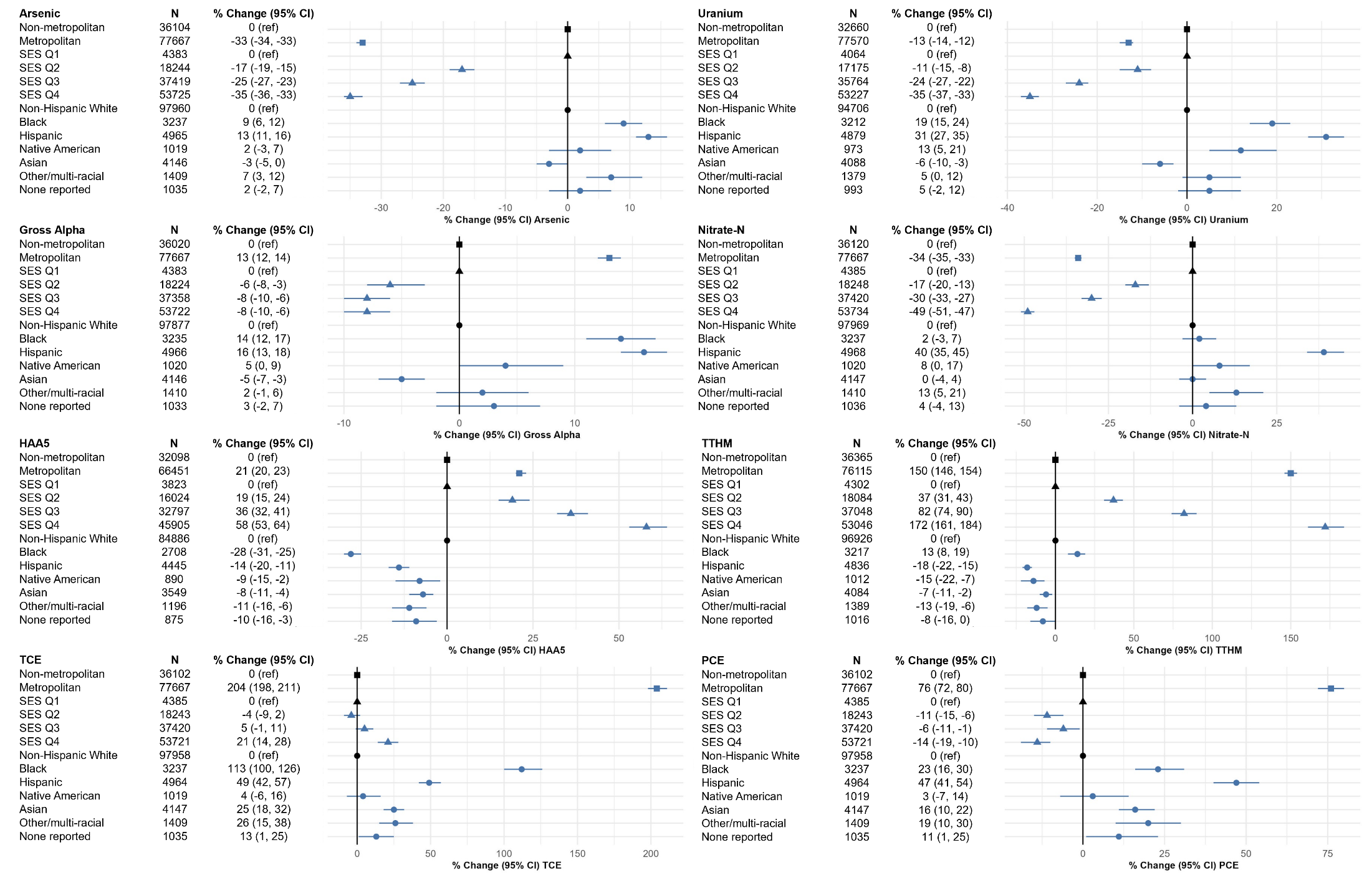


^1^Generalized linear regression was used to compute geometric mean ratios (GMRs) (95% confidence intervals, CI) of natural-log transformed exposure estimates by SES quartile (*reference=SES quartile 1),* urbanicity (*reference=non-metropolitan areas*), and participant race/ethnicity *(reference=non-Hispanic white participants)*. We calculated percent changes as: (GMR – 1) × 100%.

^2^Contaminants are as follows: arsenic (µg/L), uranium (μg/L), gross alpha (pCi/L), nitrate-nitrogen (Nitrate-N, mg/L), total trihalomethanes (TTHM, μg/L), five haloacetic acids (HAA5, μg/L), trichloroethylene (TCE, μg/L), and tetrachloroethylene (PCE, μg/L). Measurement data below the detection limit for the purposes of reporting (DLR) were imputed using Tobit regression.

^3^SES metric was created incorporating three 1990 census block group variables (occupation, education, and income) (Hurley et al. 2005).

^4^Urbanization categories were created using 1990 census block groups (Hurley et al. 2005) and dichotomized as non-metropolitan (rural, town, city), and metropolitan (metropolitan suburban and metropolitan urban).
